# A methylated lysine is a switch point for conformational communication in the chaperone Hsp90

**DOI:** 10.1038/s41467-020-15048-8

**Published:** 2020-03-05

**Authors:** Alexandra Rehn, Jannis Lawatscheck, Marie-Lena Jokisch, Sophie L. Mader, Qi Luo, Franziska Tippel, Birgit Blank, Klaus Richter, Kathrin Lang, Ville R. I. Kaila, Johannes Buchner

**Affiliations:** 1 Department of Chemistry, Technische Universität München, Lichtenbergstr. 4, 85747 Garching, Germany; 2 Institute for Advanced Study, Lichtenbergstraße 2a, 85748 Garching, Germany; 3 Soft Matter Research Center and Department of Chemistry, Zhejiang University, Zhejiang, 310027 PR China; 4 Department of Biochemistry and Biophysics, Stockholm University, SE-106 91, Stockholm, Sweden; 5 Present Address: Institut für Mikrobiologie der Bundeswehr, Neuherbergstr.11, 80937 München, Germany; 6 Present Address: NanoTemper Technologies GmbH, Flößergasse 4, 81369 München, Germany; 7 Present Address: Max Planck Institut für Biochemie, Department Molekulare Medizin, Am Klopferspitz 18, 82152 Martinsried, Germany

**Keywords:** Chaperones, Chaperones

## Abstract

Methylation of a conserved lysine in C-terminal domain of the molecular chaperone Hsp90 was shown previously to affect its in vivo function. However, the underlying mechanism remained elusive. Through a combined experimental and computational approach, this study shows that this site is very sensitive to sidechain modifications and crucial for Hsp90 activity in vitro and in vivo. Our results demonstrate that this particular lysine serves as a switch point for the regulation of Hsp90 functions by influencing its conformational cycle, ATPase activity, co-chaperone regulation, and client activation of yeast and human Hsp90. Incorporation of the methylated lysine via genetic code expansion specifically shows that upon modification, the conformational cycle of Hsp90 is altered. Molecular dynamics simulations including the methylated lysine suggest specific conformational changes that are propagated through Hsp90. Thus, methylation of the C-terminal lysine allows a precise allosteric tuning of Hsp90 activity via long distances.

## Abstract (web summary)

Methylation of a lysine residue in Hsp90 is a recently discovered post-translational modification but the mechanistic effects of this modification have remained unknown so far. Here the authors combine biochemical and biophysical approaches, molecular dynamics (MD) simulations and functional experiments with yeast and show that this lysine is a switch point, which specifically modulates conserved Hsp90 functions including co-chaperone regulation and client activation.

## Introduction

Hsp90 is a highly conserved molecular chaperone in the cytoplasm of eukaryotic cells. It is responsible for the folding, activation, and maturation of several hundred client proteins^1,2^. The main classes of Hsp90 clients include kinases^3^, transcription factors^4,5^, and E3-ligases among others^6–8^. Many of these clients play a key role in diseases like cancer, cystic fibrosis, and Alzheimer’s, making Hsp90 a promising target for therapeutic approaches^9–11^.

Hsp90 is a dimeric protein, in which each protomer consists of three domains: an N-terminal domain (NTD) containing the ATP binding pocket, a middle domain important for client and co-chaperone interaction, and a C-terminal dimerization domain (CTD)^12–14^. The dimeric protein traverses from an N-terminally open state to a closed and twisted conformation, in which the NTDs and middle domains are associated as a prerequisite for ATP hydrolysis^15–18^. Subsequently, Hsp90 regains its open, V-shaped conformation. This ATPase cycle can be modulated by a cohort of Hsp90 co-chaperones, which are only found in eukaryotes. Some co-chaperones accelerate the Hsp90 cycle, whereas others have a decelerating effect^19–21^. A further important regulatory element of the Hsp90 cycle are post-translational modifications (PTMs)^22^. PTMs are covalent modifications of specific amino acid side chains that regulate activity, localization or interactions^23–25^. Hsp90 is strongly affected by different PTMs, e.g. phosphorylation^26–28^, acetylation^29–31^, nitrosylation^32,33^, ubiquitination^34^, SUMOylation^35,36^, nitration^37^, glycosylation^38^, and methylation^39,40^. These PTMs in Hsp90 are distributed throughout the protein^23^. Some positions targeted by PTMs have been shown to represent conformational switch points where a small modification can decisively change the properties of Hsp90 in an allosteric manner^23,28,33^.

Methylation of a lysine residue in human Hsp90 by the methyltransferase Smyd2 is a recently discovered PTM^39,40^. The methylation site is located at positions K615 (Hsp90α) and K607 (Hsp90β) in the CTD of Hsp90, and the methyl moiety can be removed by the lysine demethylase LSD1^39^. Whether Smyd2 methylates both Hsp90 protomers symmetrically is not known. The mono-methylation at this position enhances the interaction of Hsp90 with the muscle protein titin, leading to a stabilization of the I-band in skeletal as well as cardiac muscle cells^40,41^. Despite the importance of the methylation of Hsp90 for muscle stability, the mechanistic effect of this modification on Hsp90 has remained elusive. Here, we set out to investigate how variations at this position affect the structure and function of yeast and human Hsp90 protein, complemented by atomistic MD simulations. We discovered that the methylation position is a switch point in Hsp90 conserved from yeast to man and specifically modulates conserved Hsp90 functions.

## Results

### The methylation position is conserved in the Hsp90 family

**Fig. 1:**
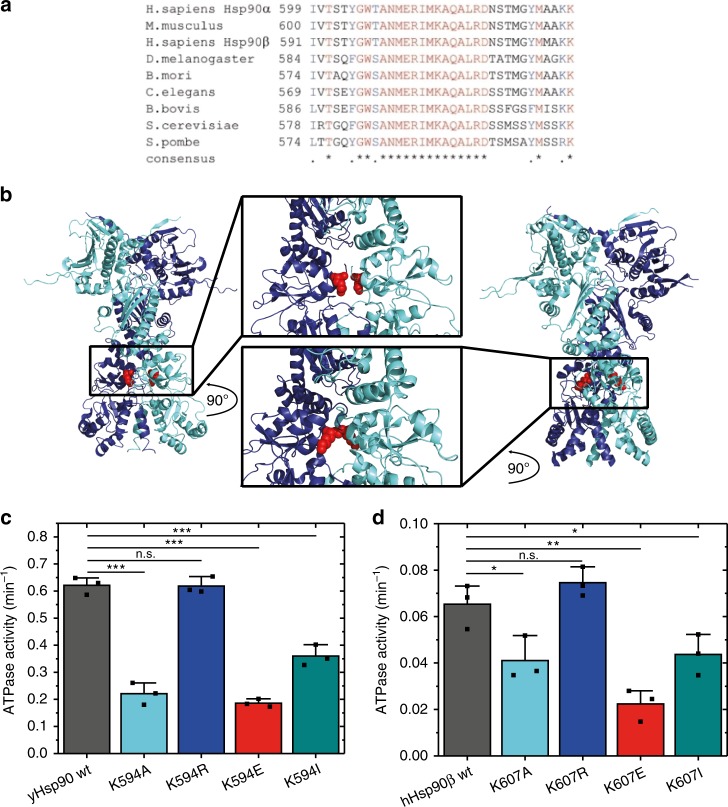
Position of the modified lysine residue and ATPase of Hsp90 variants. **a** Sequence alignment of various cytosolic Hsp90s. The alignment highlights the methylation position, which is embedded in a highly conserved amino acid stretch. **b** Crystal structure of yeast Hsp90 (left)^15^ and human Hsp90 (right)^42^ in which the conserved lysine residue (K594/K607) (red) is located in a helix. The intrinsic ATP turnover of yeast Hsp90 (**c**) and human Hsp90ß (**d**) was determined using an ATP regenerating assay. The ATPase activity of the human homologue was already shown to be significantly lower compared with the yeast version and is herein confirmed. All replacement mutants except for the substitutions from lysine to arginine resulted in an impairment of the ATP turnover. The WT is shown in black/dark grey, the alanine substitution in light blue, the arginine substitution in dark blue, the glutamate substitution in red, and the isoleucine substitution in cyan dark. The colour code is used for the mutations throughout the manuscript. The error bars for the turnover rates represent s.d. from three independent measurements (*n* = 3). Statistical significance was assessed using a two-sample *t*-test and a level of significance of 0.05 (*), 0.01 (**), and 0.001 (***). Source data are provided as a Source Data file.

Lysine methylation is a PTM of Hsp90 that has been described for both human homologues^39,40^. The respective lysine residue is part of a highly conserved amino acid sequence in eukaryotic Hsp90s (Fig. 1a). In fact, this amino acid stretch (ANMERIMKAQALRD) is the longest conserved sequence in the CTD. This supports the importance of the region and the methylation position, as the sites of other PTMs are not generally conserved^23,28^. In the crystal structure of yeast Hsp90^15^, the ANMERIMKAQALRD region is located at the intersection between a helix and an unstructured/unresolved loop. For human Hsp90ß^42^, the surrounding of K607 is similar with the difference that the region is fully resolved, showing an extended helix compared with the yeast homologue (Fig. 1b).

### Lysine mutations affect the Hsp90 ATPase

We wondered whether the position of the lysine in Hsp90 targeted by methylation is in general sensitive to changes in its amino acid sidechain. We therefore replaced the respective lysine, K594 in yeast Hsp90 (yHsp90), and K607 in human Hsp90β (hHsp90β), by four different amino acids: alanine (neutral), isoleucine (hydrophobic), glutamic acid (negative charge), or arginine (positive charge). These variants were expressed in *E. coli* and purified. The mutant proteins were stable and adopted wild type (WT)-like secondary structures (Supplementary Fig. 1A, B). Since the mutated site is close to the C-terminal dimerization surface, we first tested the influence of the introduced mutations on the dimerization properties^43^. The *K*_*D*_ values determined are in the same range (30–60 nM for yHsp90 and 6–13 nM for hHsp90β). Thus, the mutants do not exhibit significant changes in dimer formation compared with the WT (Supplementary Fig. 2A, B). Although the mutated site is far away from the ATPase site in the NTD, we tested whether the intrinsic ATPase activity of Hsp90 is altered and observed pronounced effects. For all yHsp90 mutants, except the replacement of lysine by arginine (K594R), the ATP turnover is impaired (Fig. 1c). The negative charge substitution showed the strongest effect (about 70% reduction), while the neutral and hydrophobic substitutions caused a slighter decrease. The same effects were obtained for the mutations in hHsp90β (Fig. 1d): again, the replacement of lysine with an arginine had no significant effect on the ATPase activity, whereas the neutral, hydrophobic, and negative charge substitutions caused reductions in the ATP turnover. The most severe defects were again observed for the negatively charged replacement mutant K607E. To probe whether ATP binding was also influenced by the substitutions, ATPase activity assays were performed with varying concentrations of ATP (Supplementary Fig. 3A, B). The *K*_*M*_ values of the different mutants were comparable to those of the WT, showing that the affinity for ATP is not affected by the substitutions.

These results indicate three important features: first, the ATP turnover can be regulated by changes at the position of the lysine in the CTD, ~70 Å away from the active site, and hence this residue is an allosteric regulation point. Second, a positive charge at this position is crucial for a WT-like ATPase activity, as arginine can replace lysine without affecting the ATP turnover. Third, the allosteric regulation point is conserved, as the human and the yeast homologues react identically to the amino acid changes at this position.

### Lysine mutations affect co-chaperone interaction

**Fig. 2:**
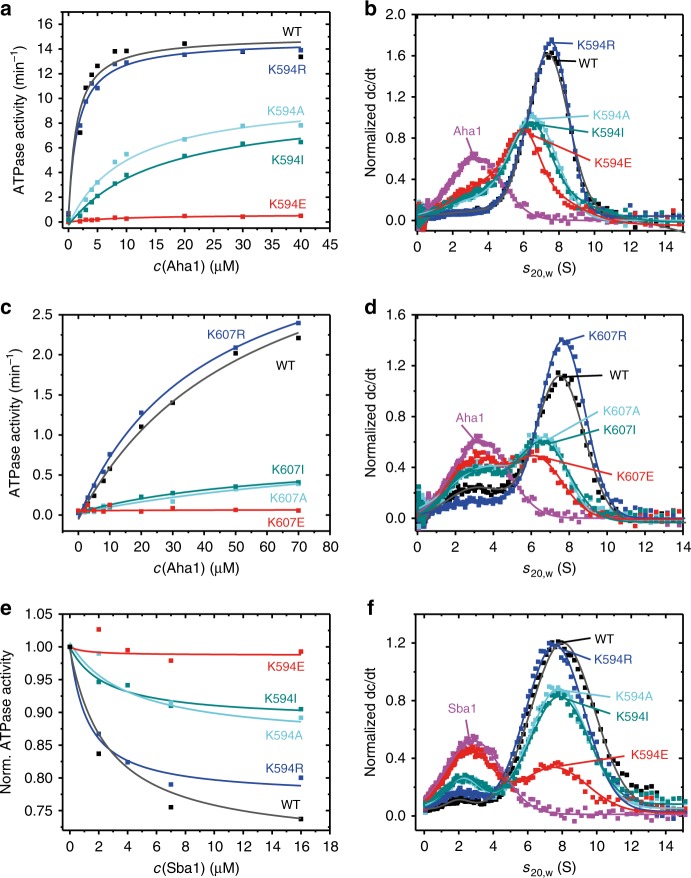
Co-chaperone actions are influenced by the modifications. **a**, **c** ATP turnover of the WT and the different mutants in the presence of the co-chaperone Aha1. **b**, **d** The binding of the different Hsp90 constructs to Aha1 was analyzed by analytical ultracentrifugation. dc/dt plots are shown for Aha1-FAM (purple) and the mixtures of Aha1-FAM with the indicated Hsp90 constructs. **e** Normalized ATPase activity of the WT and the mutants in the presence of the inhibitory protein p23/Sba1. **f** The binding of the different Hsp90 mutants to p23/Sba1 was analyzed by analytical ultracentrifugation. dc/dt plots are shown for p23/Sba1-Atto488 (purple) and its mixtures with the indicated mutants.

Some co-chaperones bind to specific Hsp90 conformations and are thus excellent tools for the analysis of the Hsp90 cycle^20,44^. As the co-chaperone Aha1 accelerates the Hsp90 ATPase activity^45^ by binding to the M- and N-domains of Hsp90^46–48^, and promoting the formation of the closed state^47,49,50^, we used Aha1 as a sensor for this conformational transition in the variants studied and performed ATPase activity assays with increasing Aha1 concentrations. In yHsp90, for the mutant K594R, the presence of Aha1 led to a similar activation as observed for the WT protein. K594A and K594I, were in contrast less activated, and K594E did not show any stimulation, even at the highest employed Aha1 concentration (Fig. 2a). For the respective variants of human Hsp90, a conserved pattern of effects concerning the ability to stimulate the ATPase by Aha1 was observed: K607R and hHsp90β-WT were stimulated comparably, whereas K607A and K607I were only accelerated by ~20% of the WT levels and, again, the K607E mutant was not activated by Aha1 (Fig. 2c). To test whether the yeast and human proteins are still able to bind Aha1, we performed analytical ultracentrifugation (aUC) experiments with FAM-labelled Aha1 (Fig. 2b, d). Complex formation by binding of Aha1 to Hsp90 results in a shift of the sedimentation coefficients from 3 to 8 s. We observed complex formation for all yHsp90 variants, with WT and K594R showing identical sedimentation profiles (Fig. 2b). For the interaction of Aha1 with K594A, K594I, and K594E, lower *S* values were observed for the complexes. This can be explained by a different shape of the complex, possibly a more open conformation. We also obtained similar results for the human proteins (Fig. 2d). K607R showed an identical sedimentation profile as the WT protein, while all other mutants exhibited a decreased interaction with Aha1 and hence a less pronounced shift in the *S* values. Importantly, the binding sites for Aha1 are in the M- and N-domains of Hsp90^46–48^. Taken together, these results suggest that alterations at this conserved lysine in the CTD lead to changes in the Aha1-supported conformational rearrangements in the M- and N-domains of Hsp90. Interestingly, while Aha1 is still able to bind to yeast and human Hsp90, no stimulation of the ATPase activity of the K594E and K607E mutants could be detected. Hence, the closing defect of these mutants cannot be rescued by Aha1.

We next probed the influence of the inhibitory co-chaperones p23/Sba1 and Sti1 on the yeast mutants^19,51,52^. The human variants were omitted from this experiment as the intrinsic ATP turnover of the human variants and the inhibitory effect of p23 and Sti1 is quite low, which makes the detection of a further decrease difficult. Recent studies however showed that some newly discovered co-chaperones are impacting Hsp90’s ATPase activity to a higher degree and might therefore be a viable sensor for future studies^21,22^. Sti1 binds to Hsp90 in its open conformation and prevents further rearrangements towards the closed state, thereby inhibiting the ATP turnover of the protein^19,53^. For all yeast variants, we observed a WT-like inhibitory effect of this co-chaperone, indicating that neither Sti1 binding nor its ability to keep the mutants in an open conformation is diminished by the substitution of the lysine residue (Supplementary Fig. 4).

p23/Sba1 binds to the NTD of Hsp90 in its closed conformation^15,54–56^, and can thus be used as a sensor to specifically probe the ability of the mutants to reach the closed-2 state^50,56^. We therefore analyzed the effect of Sba1 on the inhibition of the Hsp90 ATPase activity^51,57^ (Fig. 2e), and determined the binding of Sba1 by aUC (Fig. 2f). Binding of labelled Sba1 to Hsp90 leads to a shift in the *S* value from 3 to 8 s. All yeast mutants showed this shift although to different extents. K594R and the WT protein bound equally well to Sba1 and inhibited the ATPase activity of Hsp90 to the same level, whereas binding and inhibition of the neutral substitutions K594A and K594I were slightly impaired. K594E instead showed the lowest complex formation and no inhibition of the ATPase activity. Thus, alteration of the conserved lysine residue in the CTD affects the ability of Hsp90 to adopt a closed conformation, which can be bound by p23/Sba1.

### In vivo effects of mutations in the methylated site

**Fig. 3:**
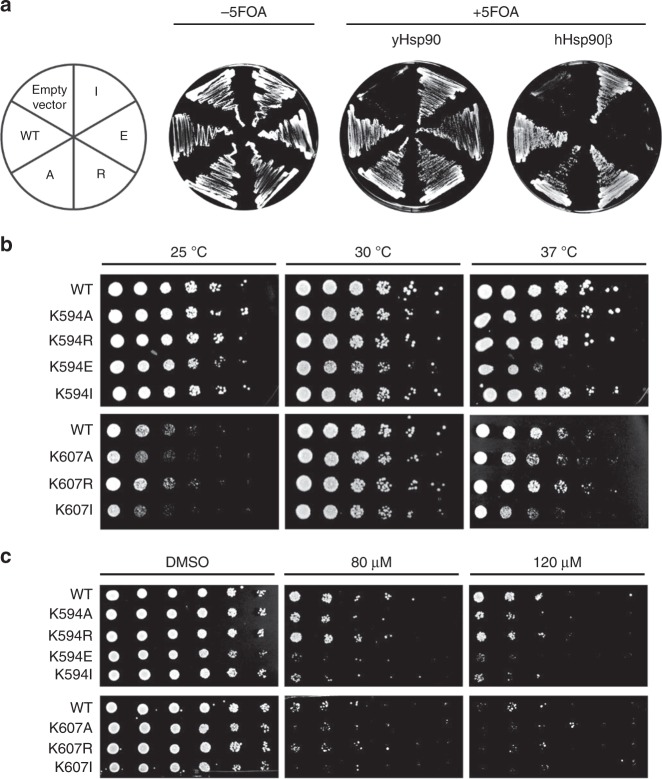
Influence of lysine variants in vivo. **a** A FOA-shuffling approach was used to introduce the Hsp90 variants as the sole source of Hsp90 in the yeast *S. cerevisiae*. All mutants, except those carrying the empty vector and the human Hsp90ß mutant K607E were viable. **b** Drop assays to observe growth differences between the different strains. All shuffled yeast strains grew similarly at 30 °C but started showing growth deficiencies at lower and higher temperatures. **c** Hsp90 mutants were tested towards their sensitivity against various concentrations of the inhibitor radicicol. Source data are provided as a Source Data file.

To test whether the variants affect the function of Hsp90 in vivo, we introduced them as the sole source of Hsp90 in *S. cerevisiae*. It was previously described that also the human isoforms can substitute the essential function of Hsp90 in yeast and confer viability^58^. We observed that all yeast and human mutants, except for human K607E, which had already shown the most severe in vitro defects, support yeast viability (Fig. 3a). Since human Hsp90 cannot replace yeast Hsp82 efficiently^58^, the presence of a mutation that affects Hsp90 function may therefore be more severe for the human protein, especially in the yeast viability assay. At the optimal growth temperature of 30 °C for *S. cerevisiae*, the negative charge mutant K594E grows slower as compared with the WT and the other replacement mutants (Fig. 3b). When the mutants were shifted to higher (37 °C) or lower (25 °C) temperatures, K594E differed even more in its growth behaviour. The human variants reacted more sensitive to a change in temperature, independently of the direction of the shift. At these temperatures, the growth of the hHsp90ß-WT was already impaired compared with the yeast WT. The neutral substitution mutants, K607A and K607I, displayed even greater growth deficiencies. Only K607R showed a growth rate similar to its corresponding WT (Fig. 3b).

We next tested the effect of the Hsp90 inhibitor radicicol towards the yeast mutants, as it is known from previous studies that changes in the M- and C-domains can lead to differences in radicicol sensitivity^28^. Radicicol competes with ATP for binding to the Hsp90 NTD^59,60^. It affects both the yeast and the human Hsp90 homologues^58,61^. Hence, all cells were grown in the presence of different radicicol concentrations and subsequently spotted on agar plates (Fig. 3c). For the yeast proteins, only K594R showed a WT-like sensitivity, while the alanine and isoleucine mutants exhibited an increased sensitivity for the inhibitor. Yeast strains carrying the K594E mutant were barely viable in the presence of radicicol. The same observation was made for the yeast strains carrying the human Hsp90 homologues (Fig. 3c, lower panel; for quantification see Supplementary Fig. 5). There was only marginal growth for any strain, even the one carrying Hsp90ß-WT. Taken together, these results show that lysine 594/607 is important for the in vivo function of Hsp90 in yeast.

### Changes in the methylation position affect client processing

The results described so far demonstrate that changes at the position of the methylated lysine allosterically modulate Hsp90 in vitro and in vivo. We next addressed how changes at this position affect client protein activation in *S. cerevisiae* using selected client proteins for which well-established assays exist^28,62,63^.

**Fig. 4:**
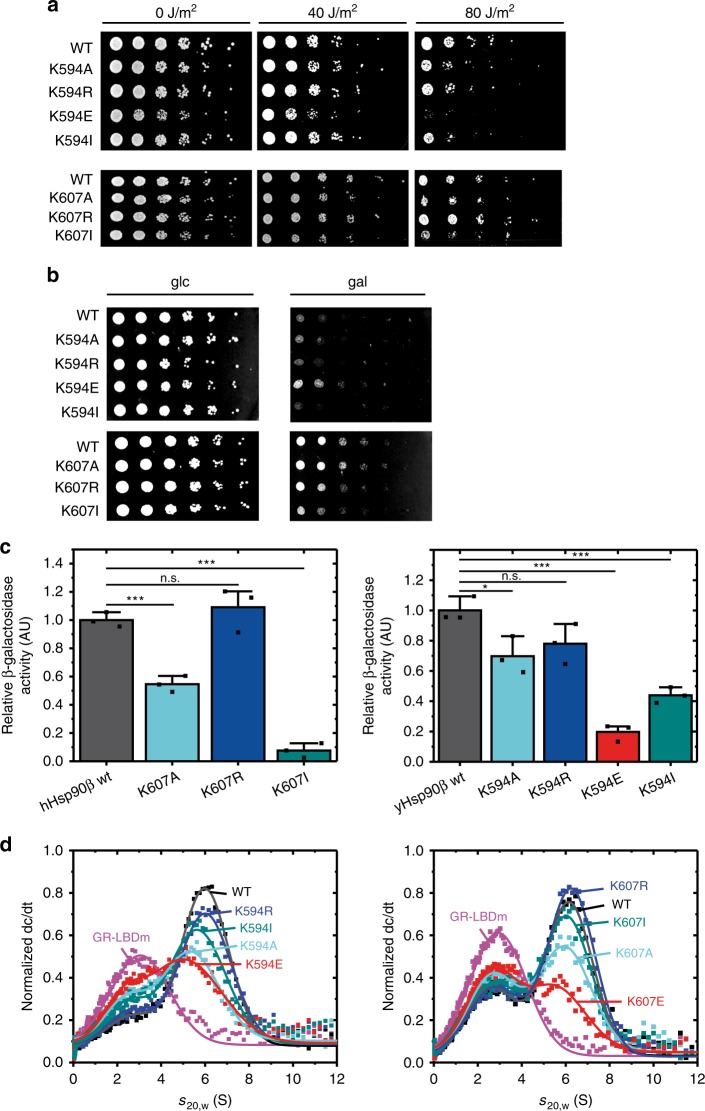
Changes at the position of the conserved lysine affect client activation. **a** Yeast strains were exposed to the indicated dosage of UV light and subsequently analyzed for their ability to induce the Hsp90 dependent nucleotide excision repair. **b** The activation of the Hsp90 client v-src was tested by a lethality assay. Survival implies the inability of Hsp90 to activate v-src. **c** The activity of the glucocorticoid receptor (GR), which is dependent on Hsp90 was measured in a ß-galactosidase assay. Activities were normalized to ß-galactosidase activity of wt Hsp90. The error bars for the activity represent s.d. from three biological replicates (*n* = 3). Statistical significance was assessed using a two-sample *t*-test and a level of significance of 0.05 (*), 0.01 (**), and 0.001 (***). **d** The binding of the different Hsp90 constructs to the ligand binding domain of the glucocorticoid receptor (GR-LBD) was analyzed by analytical ultracentrifugation. dc/dt plots are shown for GR-LBD-Atto 488 (purple) and the mixtures of GR-LBDm-Atto 488 with the indicated Hsp90 mutants. Source data are provided as a Source Data file.

Several components of the endogenous yeast DNA repair mechanism were previously described as Hsp90-dependent^64–66^. Exposure of yeast cells to different dosages of UV-light leads to pyrimidine dimer formation, and in consequence to induction of the Hsp90 dependent nucleotide excision repair^67^. At a light dosage of 40 J/m^2^, only a slight growth deficiency was observed for the yeast carrying the K594E mutant, while the other mutants showed WT-like behaviour (Fig. 4a). At 80 J/m^2^, the differences were more pronounced: K594E could not recover from the applied stress and the yeast carrying the alanine and isoleucine mutants were more affected than their corresponding WTs or the arginine replacement.

v-Src kinase is a strictly Hsp90-dependent client. When expressed in yeast in its active form, v-Src leads to hyper-phosphorylation and subsequent cell death^68^. This effect directly depends on the function of the Hsp90 system^62^, and the mutants can thus be easily tested by a viability assay. We found that all yeast variants, except K594E, were able to activate v-Src (Fig. 4b). In contrast, yeast carrying the human Hsp90ß-WT and the respective human mutants were not able to fully activate v-Src, as cell growth was still detectable. The results for hHsp90β are in agreement with previous data, where yeast, expressing hHsp90β, were shown to be less sensitive to the lethal effects of v-Src^58^.

We next assayed the activity of another stringent client of Hsp90, the glucocorticoid receptor (GR) in the presence of the Hsp90 mutants, using a β-galactosidase-based reporter assay^63,69^ (Fig. 4c). We observed an activity pattern for the different mutants comparable to that obtained in the assays above. The substitution of the lysine to the arginine (K594R/K607R) resulted in a WT-like activation of the GR. The substitution to glutamate (K594E) exhibited an 80% reduction of GR activation, while the substitutions to alanine and isoleucine (K594A/K607A and K594I/K607I) lead to a decrease in the activation of about 50%. For human Hsp90β, the activation of GR by K607I was slightly more decreased.

Taken together, changes at the position of the conserved lysine affect client processing in the living cell and are thus crucial for the biological function of Hsp90.

In addition, aUC experiments were performed using the purified GR-LBD as a client in vitro (Fig. 4d). The results show an impaired binding of the GR-LBD to the respective Hsp90 mutants with K594E and K607E exhibiting the strongest effects. The results are in line with the in vivo data and demonstrate the importance of lysine 594/607 on regulating client interaction.

### Molecular dynamics simulations of Hsp90 variants

**Fig. 5:**
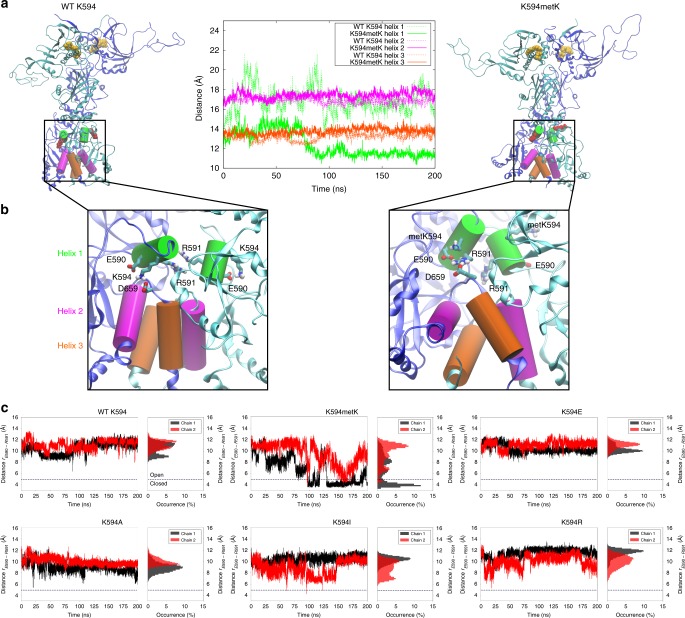
Molecular dynamics simulations of the Hsp90 variants. **a**, **b** Snapshots obtained from 200 ns MD simulation of the wild type (WT) and mono-methylated Lys-594 model (metK594). ATP, located in the N-domain, is shown in van-der-Waals (vdW) representation (in yellow). The constitutive interface of the two C-domains comprises three pairs of helices, with K594/metK594 located on Helix 1 shown in vdW representation (in red). The figure shows the distance between the centres of mass of three pairs of helices from the two CTDs. **c** The distance and probability distribution (occurrence, %) of the E590-R591 (Cδ-Cζ) ion pair in MD simulations. Open (*r* > 5 Å) and closed (*r* < 5 Å) conformations of the ion pair are marked with a dashed line. In WT Hsp90, the ion pair remains open in both Hsp90 protomers (chains 1 and 2), whereas the ion pair undergoes several closing transitions upon methylation. In terms of these ion-pair dynamics, the K594I simulation resembles the methylated Lys-594 simulation, with a few closure events observed during the trajectory.

To probe how PTMs and residue substitutions at the position of the conserved lysine influence the dynamics of Hsp90, we performed atomistic molecular dynamics (MD) simulations of the mono-methylated Lys-594 (K594metK), as well as for the in silico mutated K594R/E/I/A constructs and compared them with the dynamics of yHsp90-WT. All models were constructed based on the X-ray structure of the full-length yeast Hsp90 dimer (PDB ID: 2CG9)^15^. We observed that in the simulations with a methylated Lys-594, the distance between a pair of helices of the CTDs decreases in comparison to WT-Hsp90 (Fig. 5a, b) and the number of salt bridges between the two domains is slightly increased (Supplementary Fig. 6A–F). Interestingly, the dimeric pair of helix-1 in the CTD, which forms a putative dimerization interface in Hsp90^15^, moves closer together and causes conformational changes in the surrounding charged residues (Fig. 5a, b). For the non-methylated WT-Hsp90 model, Lys-594 strongly interacts with Glu-590 and Asp-659, whereas the methylation increases the distance between these residues (Fig. 5a, b). This in turn enhances the interaction between Glu-590 and Arg-591, thus increasing the inter-subunit contacts (Fig. 5c; Supplementary Fig. 6A, B). When Lys-594 is modelled in its methylated state, Arg-591 takes the position of the lysine sidechain, which in turn results in more closely packed helices at the dimerization interface. Moreover, Arg-599 and Asp-600, which are located in the loop following helix 1 with the methylated Lys-594, make contacts with Glu-431 and Lys-335 from the middle domains, leading to the formation of two new salt bridges between the CTD and the middle domains (Supplementary Fig. 7)

In order to identify if the studied single point substitutions, K594R/E/I/A, resemble the effect of the methylated Lys-594, we performed atomistic MD simulations on the in silico mutated constructs and analyzed their ion-pair dynamics (Fig. 5c). We found that in WT-Hsp90 simulations, the Glu-590/Arg-591 ion pair remains open, whereas the methylation triggers several closing transitions in this ion pair, indicating that the PTM modifies the free energy landscape of the dimerization interface. Interestingly, we found that in simulations of the K594I, the Glu-590/Arg-591 also flickers between open and closed states, suggesting that K594I could mimic, at least in part, the effect of Lys-594 methylation.

Although the simulation timescale is here limited to a few hundred nanoseconds due to the large size of the protein, our analysis of ion-pair dynamics and inter-domain salt bridges indicates that the methylation or mutation of Lys-594 may also affect the conformational dynamics in the global Hsp90 structure (Supplementary Figs. 6A–F and 8A–D). The K594I model shows in addition to an increase in ion pairs between the CTDs, also a slight increase in salt bridges between the two middle domains (Supplementary Fig. 6A–F). Moreover, we found that the dynamics of the helices at the dimerization interface resemble each other in the methylated lysine and K594I models (Supplementary Fig. 9), further supporting that K594I could mimic the effect of Lys-594 methylation. Interestingly, all Lys-594 substitutions show a small decrease in the ion pairs between the two NTDs as compared with WT-Hsp90 (Supplementary Fig. 6A–F). For example, the MD simulation of the WT protein shows a strong intermolecular ion pair between Glu-11 and Lys-102 of the two NTDs that is absent in the monomethylated variant, indicating some conformational changes that could propagate across the Hsp90 dimer (Supplementary Fig. 7). These findings are consistent with our previous experiments showing that modifications at this position have a significant influence on NTD dimerization and therefore serve as an allosteric regulation point in Hsp90. Moreover, some global conformational changes in the dynamics are also supported by analysis of backbone root-mean-square deviations (RMSD) (Supplementary Fig. 10). Importantly, although the primary events leading to the global dynamical changes in Hsp90 are probably captured during the MD trajectories, the overall simulation timescales is, nevertheless, too short to accurately probe the global conformational changes in Hsp90 structure. Overall, however, our MD simulations suggest that methylation of Lys-594 influences the C-terminal domain by affecting electrostatic interactions amongst charged residues. The combined electrostatic-conformational effect may propagate further to the middle- and N-terminal domain, supporting the putative role of Lys-594 as an important switch point in Hsp90.

### Replacement of the lysine causes structural changes in Hsp90

**Fig. 6:**
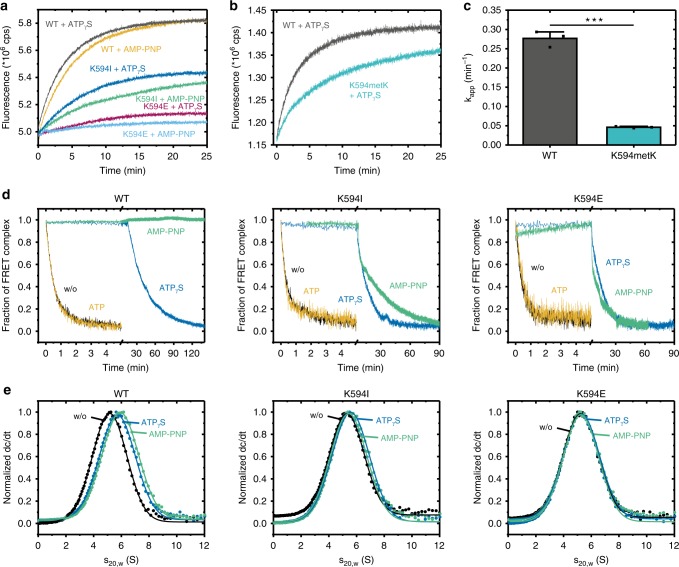
Comparison of the conformational rearrangements in the Hsp90 variants. **a** The nucleotide-induced closure of the FRET pair labelled dimer was monitored in the acceptor channel after addition of the different nucleotides. **b** ATPγS induced closing kinetics of mono-methylated Hsp90 (K594metK) and WT yeast Hsp90. **c** Closing kinetics of three independent measurements. Data were fitted using a bi-exponential fit (see methods Eq. 1). The error bars for the turnover rates represent s.d. (*n* = 3). Statistical significance was assessed using a two-sample *t*-test and a level of significance of 0.05 (*), 0.01 (**), and 0.001 (***). **d** The disruption of a preformed FRET complex was followed after adding an excess of unlabelled protein. Left panel shows the kinetics for the WT and the middle and right panel for the K594I and K594E mutants. **e** Sedimentation profiles of the labelled WT, K594I, and K594E mutants in the absence and presence of the nucleotides ATPγS and AMP-PNP. Source data are provided as a Source Data file.

To directly test the effect of lysine methylation, we set out to introduce a mono-methylated lysine at position 594 in yeast Hsp90 via amber suppression (K594metK) using orthogonal *Methanosarcina barkeri* pyrrolysyl tRNA synthetase (PylRS)/tRNA pairs (Supplementary Fig. 11A). We screened different PylRS mutants for efficient and site-specific incorporation of Nε-methyl-Nε(p-nitrocarbobenzyloxy)lysine (met-ncbK) into proteins bearing a prematurely introduced amber codon (Supplementary Fig. 11B). A recently discovered PylRS variant showed efficient production of full-length proteins and site-specific and quantitative incorporation of Met-Lys (metK), determined via LC–MS (Supplementary Fig. 11B). We were able to express and purify mono-methylated Hsp90 (Supplementary Fig. 11C). MS analysis revealed a 30% incorporation of metK resulting in a distribution of WT/WT (0.7 × 0.7 = 0.49), WT/K594metK (0.3 × 0.7 × 2 = 0.42), and K594metK/K594metK (0.3 × 0.3 = 0.09) dimer formation resulting in a total of 51% methylated species. For fluorescence resonance energy transfer (FRET) experiments, we labelled the three mutants (K594I, K594E, K594metK) at specific sites in the N-domains with donor and acceptor dyes and analyzed their conformational changes^16^. In the presence of nucleotides, the WT Hsp90 as well as the K594I, K594E, and K594metK mutants showed an increase in the acceptor fluorescence, which is indicative of the formation of a closed state. However, we found that the kinetics and amplitudes of the closing process are significantly decreased for the mutants, with K594E having the strongest effect (Fig. 6a, b). The addition of the non-hydrolyzable ATP analogue, AMP-PNP, gave an even stronger effect. Interestingly, the K594metK mutant showed similarities in regards of a decline in the amplitude as well as a slower closing, compared with K594I upon ATPγS addition. We found that the kinetics of N-terminal dimerization are significantly impaired compared with the WT (Fig. 6c). The lack of closed conformations is in agreement with the lowered ATPase activity, the defect in p23/Sba1 binding, and the somewhat lowered number of salt bridges formed between the NTDs in the MD simulations. We next tested the stability of the dimeric complex in the presence of various nucleotides, which reports on the presence of N-terminally closed states (Fig. 6d). To this end, an excess of unlabelled protein was added to a preformed FRET-hetero complex. In the absence of nucleotides and in the presence of ATP, for the WT as well as the K594E and K594I mutants, FRET dimers could be rapidly dissociated by the excess of unlabelled protein. This effect arises from the open conformation of the FRET-hetero complexes in which subunit exchange can rapidly occur^16,70^. In the presence of ATPγS, a disruption of the WT and mutant complexes was also observed, but for K594E and K594I, the dissociation was significantly faster, which is consistent with the slower closing kinetics (Fig. 6a). The largest differences were monitored in the presence of AMP-PNP. For K594E, a rapid dissociation of the complex was observed after addition of unlabelled Hsp90. For K594I, the dissociation was slightly slower, whereas the WT remained in its closed conformation. These results support the notion that the N-terminal dimerization is impaired in the mutants.

In addition to the FRET experiments, we also performed aUC for the WT protein and the K594I and K594E mutants in the presence and absence of nucleotides (Fig. 6e). In these experiments, a shift towards higher *S* values corresponds to a more compact conformation of the Hsp90 dimer. For the WT protein, we observed a shift towards higher *S* values in the presence of ATPγS or AMP-PNP, whereas the K594I mutant showed significantly lower *S* values (Supplementary Table 1). Furthermore, the K594E mutant did not exhibit a detectable shift, neither in the presence of ATPγS nor AMP-PNP. These results are in line with the closing kinetics (Fig. 6a) and chasing kinetics (Fig. 6d) determined by FRET, demonstrating a defect in the N-terminal dimerization of both mutants, with K594E showing a more pronounced effect.

## Discussion

The eukaryotic Hsp90 is subjected to various PTMs. Some transient modifications regulate the association or dissociation of co-chaperones as well as client protein maturation^28,30,33,35^. However, the effect of the recently discovered methylation of Hsp90 by the lysine methyltransferase Smyd2^39,40^, which is required for muscle stability^40,41^, has remained elusive. We set out to analyze the effect of variations at this conserved lysine position in vitro and in vivo and found that the site functions as an allosteric regulation point in Hsp90.

The exchange of the lysine by amino acids differing in size, hydrophobicity, and charge caused pronounced effects in Hsp90. Especially the introduction of a negative charge resulted in functional consequences in distinct parts of the protein. Strikingly, the intrinsic ATP turnover of Hsp90 was affected. This is interesting as the site is located in the CTD and ~70 Å away from the ATP binding site in the NTD and the catalytic loop in the M-domain. There are two major ways in which PTMs can change the function of a protein, either via a direct mechanism at the site of the modification or by changing the protein conformation. In the latter, the effects are allosteric as part of the protein far away from the modified amino acid is influenced^71^. We refer to these specific amino acids as “switch points” as these respond to small changes and have a huge impact on the overall conformation and function of the protein. These sites are often targeted by PTMs.

All studied single point mutants, except for the lysine to arginine substitution, resulted in an impaired ATPase activity. Comparing the replacement by sidechains of different character, negative charges revealed the most severe effects. It was recently suggested by MD simulations that ATPase-inhibiting allosteric regulation points rigidify the movements of the N- and M-domains, thereby decelerating the formation of the hydrolysis-competent, closed state^72^. The same could be envisioned for these mutants. In the case of the negative charge replacement, our FRET and aUC data show that the formation of the N-terminally closed state is impaired. Accordingly, the conformation-sensitive co-chaperone p23/Sba1, a known sensor of the N-terminally closed state^51,54,56^, exhibited decreased binding to the neutral and reverse charge mutants compared with the WT. Interestingly, the co-chaperone Aha1, which accelerates the formation of the closed state in the WT protein^13,50^, could not influence the kinetics and thus was not able to overcome the conformational restrictions caused by the amino acid replacements. Since Aha1 binds to the M- and N-domains of Hsp90, a direct effect on its binding can be excluded. With regards to client protein binding, the ligand binding domain of the GR (GR-LBD), which is known to bind to the closed conformation of Hsp90^73^, showed a decreased affinity for the neutral and positively charged substitutions.

The open state however seems not to be influenced by any of the tested mutations, as we showed that the binding of the co-chaperone Sti1, which is known to bind to Hsp90 when adopting an open conformation is unchanged^19,53^. Further, aUC experiments demonstrate no changes in sedimentation in the absence of nucleotide.

The defects in forming N-terminally closed states and the concomitant changes in timing of specific steps of the conformational cycle translate into function defects in client interaction and activation as shown in vivo and in vitro. Thus, modulation of this residue by PTM seems to allow fine-tuning of Hsp90 towards client processing.

In contrast to functionally important amino acids, PTM positions are generally not conserved^23,28^. There are some positions that cannot be found in different organisms, while others seem to be more conserved. In the case of the methylation positions, a highly conserved structural element is targeted. This high invariance is also reflected in the identical effects of the amino acid substitutions in the yeast and the human proteins. The analysis of both proteins in parallel showed that they react identically to the changes at the position of the methylated lysine. This is in agreement with previous studies which showed that the basic principles of Hsp90 function are conserved^74,75^. Replacing the lysine by neutral amino acids such as alanine or isoleucine resulted in similar effects in yeast and human Hsp90 in all tested assays. Both substitutions decreased the ATPase activity to about 50% of the WT activity, and led to an impaired interaction with the co-chaperones p23/Sba1 and Aha1 as well as the GR-LBD. Also, the reverse charge replacements to a glutamic acid had a severe effect in both the yeast and human proteins. Notably, the effect of the glutamate substitutions on the conformation of the mutants was so pronounced that Aha1 could not impact the ATPase activity, although its binding was not inhibited.

At a first glance, the results in *S. cerevisiae* seem to differ between the yeast and human proteins, but this effect could arise from an ineffective replacement of the human Hsp90 by the authentic protein in *S. cerevisiae*, and hence the yeast cells reacted more sensitively to the replacement in the human protein. Nevertheless, the general effects of the replacement mutants remained similar. While yeast cells carrying K607E were not viable, the K594E cells were most sensitive in all stress assays tested. The neutral replacement mutants of human Hsp90 were sensitive to mild stressors like temperature changes, while their yeast homologues required more severe stressors like radicicol to induce similar effects, and similar effects were also observed in the client maturation assays. Based on our functional analysis we speculate that a symetrical methylation of both protomers might be possible since methylation induces a shift to the open conformation, which could further facilitate the accessibility of SMYD2.

There are two known phosphorylation sites in yeast Hsp90 (S602 and S604)^28^ that are in proximity to K594. However, the effects of each modification is distinct: The phosphomimetic mutant S604E influences yeast growth but has no impact on N-terminal association, whereas S602E neither affects yeast viability nor N-terminal dimerization^28^. In human Hsp90, there are three known phosphorylation sites in proximity to K607/615. Further, K607/615 is also subject to acetylation and ubiquitination^76^, demonstrating that this region is a hot spot for regulation by PTMs. We therefore speculate that this area serves as a general regulation site, allowing the fine tuning of Hsp90 for specific needs.

Atomistic molecular dynamics (MD) simulations of WT and methylated Lys-594 variant of Hsp90 suggest that the lysine methylation introduces specific conformational changes in the protein structure. We observed that the bulkier sidechain of the methylated Lys-594 leads to a decrease in the distance between two CTDs in comparison to WT Hsp90, an effect that also propagated to the M-large domain. We could track some of these effects to specific ion-pair dynamics, e.g. to the Glu-590/Arg-591 ion pair, which is perturbed due to methylation. Moreover, we identified a change in the specific interactions of the N- and M-domains and could hence show a propagation of information from the C- to the N-terminus. To this end, our simulations of the K594I variant showed similar behaviour as the methylated Lys-594 variant of Hsp90, suggesting that the K594I variant might resemble the effect of lysine methylation. We therefore think that the effects we saw for the K594I mutant in vitro and in vivo represent those induced by the methylation of lysine 594/607. Our findings additionally imply that the substitution to an isoleucine might be able to mimic the effects of a mono-methylated lysine in general. Overall, our MD simulations suggested that Lys-594 methylation perturbs both intra- and inter-molecular electrostatic interactions between charged residues in the CTD, the middle domain, and with some effects also visible in the NTD, supporting that Lys-594 has an important role as a switch point in Hsp90. There are other known switch points in Hsp90. Retzlaff et al. discovered that substitutions at C597 (human Hsp90) or A577 (yeast Hsp90) both affect C-terminal as well as N-terminal dimerization^33^. In human Hsp90, C597 is thought to be nitrosylated by the endothelial nitric oxide synthase, which is regulated by Hsp90^77^. Rutz et al. showed that mutations at position W312 (human Hsp90)/W300 (yeast Hsp90) resulted in a more closed conformation of Hsp90 with an increased ATPase activity and a deficiency to maturate the GR^78^. In this work, a switch point was identified, which affects N-terminal dimerization, and ATPase activity.

We speculate that the respective switch points may have specific effects on different clients and adjust Hsp90 in a certain way. A cross talk of the Hsp90 switch points might add another layer to the regulation.

There are different models to explain our results. Hsp90 cycles through energetically equivalent conformations. The binding of nucleotides shifts this equilibrium towards the N-terminally closed state. The methylation of K594/607 might cause local changes, which selectively stabilize the open conformation of Hsp90. This model of differential stabilization has been described previously in the context of different proteins^79–81^. In the case of Hsp90 methylation at K594/607 it can explain our findings regarding the closing defects, ATPase impairment, and deficiencies in the binding of co-chaperones and clients, which favour the closed conformation of Hsp90. Interestingly, however, the co-chaperone Sti1 did not show any changes in inhibiting the ATPase activity of the different variants. Further, AUC experiments of the K594I and K594E mutants showed no changes in their *S* values in the absence of nucleotides, but a shift to lower *S* values was detected in the presence of nucleotides compared with the WT. These results indicate that methylation at position K594/607 does not stabilize a specific Hsp90 conformation per se but inhibits the proper closing upon nucleotide binding. The MD simulations further suggest that the methylation affects residues in the vicinity of the modification but also in distant parts of the protein. Thus, in our view, these findings can be best explained assuming a network of inter-domain conformational communication, which is affected by methylation of K594/607. This network involves residues in the C-domain which transfer the information to parts of the M- and probably N-domain responsible for the formation of the closed state upon nucleotide binding^14,28,33,70,72,82^

Hsp90 is important for the maturation and regulation of a large number of client proteins, which do often differ in their function, structure, and interaction with Hsp90^1–8,83^. Co-chaperones and post-translational modifications can shape the landscape of these interactions in favour of a specific client protein, allowing regulation of proteostasis in a context-specific manner^19,20,23,28,33^. The muscle protein titin whose interaction with the Hsp90 machinery is affected by methylation, is an example in case, leading to a regulated arrangement of the muscle architecture^40,41^. Interestingly, in vivo studies suggested an enhanced binding of the muscle protein titin, while in our study we showed a deficit in binding the GR. We hypothesize that titin might prefer an open conformation of Hsp90 for proper interaction as it was shown for other clients^84^. The methylation of smyd2 might therefore enable Hsp90 to distinguish between different clients. The changes in Hsp90 dynamics and interaction pattern with co-chaperones or clients upon regulated changes in lysine 594/607 thus serve to adapt the chaperone to specific requirements and connects it to cellular signalling.

## Methods

### Site-specific incorporation of met-ncbK

Met-ncbK (N6-methyl-N6-(((4-nitrobenzyl)oxy)carbonyl)-L-lysine) was purchased from AF ChemPharm at >97% purity. The aminoacyl-tRNA synthetase (aaRS), which site specifically incorporates met-ncbK, was found after screening ~100 aaRS mutants available in the lab. If cells carried an aaRS mutant, which lead to amber suppression, sfGFP(N150TAG)-His_6_ was purified. First, the cells were lysed in 10 mL GFP wash buffer (20 mM Tris-HCl pH 8, 300 mM NaCl, 30 mM imidazole) per 1 g pellet using the Branson Ultrasonics™ Sonifier™ SFX250/SFX550 Cell Disruptor (20% intensity, 50% pulse, 5 min). After the lysate was cleared (14,000 × g, 15 min, 4 °C) the supernatant was incubated with 200 µL Ni2+-NTA beads (Jena Bioscience) per 1 g cell pellet for 1 h at 4 °C. Finally, the beads were washed three times with GFP wash buffer before the protein was eluted twice with 1 mL GFP elution buffer (20 mM Tris-HCl pH 8, 300 mM NaCl, 300 mM imidazole). The incorporation and decaging of the UAA was confirmed using LC–MS and SDS-PAGE. The positive hit will further be dubbed as metK-RS. This aaRS also incorporates several other unnatural amino acids^85,86^.

### Protein expression and purification of metK bearing proteins

The proteins sfGFP(N150TAG)-His_6_, myoglobine(S4TAG)-His_6_, Rab1b(Q76A, R79TAG)-His_6_, and GFP-nanobody (R18TAG)-His_6_ were expressed overnight at 37 °C in 2xYT with the respective antibiotics. At OD 0.6–0.7, protein expression was induced using 0.02% (w/v) arabinose. The medium was further complemented with 1 mM met-ncbK.^85^

sfGFP and myoglobine were purified as described above. GFP-nanobody was also purified as described above using the respective wash and elution buffers (nanobody wash buffer: 1× PBS, 500 mM NaCl, 30 mM imidazole, 2 mM PMSF; nanobody elution buffer: 1× PBS, 500 mM NaCl, 300 mM imidazole). The same purification protocol was used for Rab1b, although the respective wash buffer was complemented with 0.2 mM PMSF, 0.01 mM GDP, cOmplete protease inhibitor cocktail tablets (Roche) for cell lysis (Rab1b wash buffer: 40 mM HEPES pH 8, 2 mM ß-ME, 1 mM MgCl_2_, 500 mM LiCl, 0.01 mM GDP, 20 mM imidazole; Rab1b elution buffer: 40 mM HEPES pH 8, 2 mM ß-ME, 1 mM MgCl_2_, 500 mM LiCl, 0.01 mM GDP, 300 mM imidazole).^85,87^

The incorporation of met-ncbK and quantitative decaging to metK was confirmed using full-length ESI-LC MS (Phenomenex Jupiter^TM^ C4 column (2 × 150 mm, 5 μm)) and carried out on an Agilent Technologies 1260 Infinity LC–MS system with a 6310 quadrupole spectrometer. Buffer A was 0.1% formic acid in water; buffer B was 0.1% formic acid in acetonitrile.

### Protein expression and purification

All yeast and human mutations were introduced by site-directed mutagenesis in pET28 vectors (Invitrogen, Karlsruhe, Germany) containing the yeast (Hsp82) or human sequence of Hsp90 (Hsp90β) (Supplementary Table 2). For amber suppression, the Hsp90 sequence was introduced into a pPylT vector, encoding for the orthogonal PylT-tRNA^85^. The plasmids were transformed into BL21 (DE3) cod+ (Stratagene, La Jolla, USA) and proteins were expressed for 4 h at 37 °C (yeast) or overnight at 20 °C (human). Cells were lysed using a cell disruption system (Constant Systems, Warwick, UK). After lysate clarification, the supernatant was loaded on a 5 mL HisTrap HP column (GE Healthcare), equilibrated with Ni-NTA buffer A (40 mM KPO_4_, pH 8.0, 400 mM KCl, 6 mM imidazole). After washing the column with washing buffer (40 mM KPO_4_, pH 8.0, 400 mM KCl, 20 mM imidazole), the protein was eluted with Ni-NTA buffer B (40 mM KPO_4_, pH 8.0, 400 mM KCl, 300 mM imidazole). Fractions were pooled and diluted with Resource Q buffer A (50 mM Tris, pH 8.0, 20 mM KCl) and loaded on a 6 mL Resource Q column (GE Healthcare). Proteins were eluted with a gradient, using Resource Q buffer B (50 mM Tris, pH 8.0, 1000 mM KCl). A Superdex 16/60 200 pg column (GE Healthcare) was used for the last purification step, equilibrated with 40 mM HEPES, pH 7.5, 150 mM KCl, 5 mM MgCl_2_. For the human proteins and the Hsp82 D61C variant, 1 mM DTT was added to each buffer. For Aha1, protein expression was conducted over night at 18 °C. After the Ni-NTA (buffer A: 50 mM NaHPO_4_, pH 7.8, 300 mM NaCl, 20 mM imidazole; buffer B: 50 mM NaHPO_4_, pH 7.8, 300 mM NaCl, 300 mM imidazole), fractions were pooled and loaded directly on the Superdex column. The GR-LBD was expressed and purified according to Lorenz et al.^73^. Expression was conducted over night at 18 °C in ZYM-5052 media, supplemented with 500 µM dexamethasone (Sigma Aldrich). After cells were harvested, a PBS wash was applied and cells were lysed via sonication (Bandelin Sonopuls UW2200) in Ni-NTA buffer A (50 mM Tris, pH 7.9, 2 M Urea, 100 mM NaCl, 5 mM MgCl_2_, 10 mM imidazole, 2 mM β-mercaptoethanol, 50 µM dexamethasone). After clearance, the lysate was loaded on a 5 mL HisTrap HP column, equilibrated with Ni-NTA buffer B (50 mM Tris, pH 7.9, 500 mM NaCl, 10 mM imidazole, 2 mM β-mercaptoethanol, 10% glycerol, 50 µM dexamethasone). Proteins were eluted with Ni-NTA buffer C (50 mM Tris, pH 7.9, 500 mM NaCl, 350 mM imidazole, 2 mM β-mercaptoethanol, 10% glycerol, 50 µM dexamethasone). Fractions were pooled, supplemented with His_6_-tagged TEV-protease, and dialyzed against 50 mM Tris, pH 7.9, 100 mM NaCl, 10% glycerol, 2 mM β-mercaptoethanol, 0.5% CHAPS, 50 µM dexamethasone over night at 4 °C. The digested protein was loaded on a HisTrap HP column and the flowthrough was collected. The protein was concentrated and loaded on a Superdex 16/60 200 pg column equilibrated in 25 mM Tris, pH 7.9, 100 mM NaCl, 10% glycerol, 2 mM DTT, 50 µM dexamethasone.

For the incorporation of met-ncbK, the pPylT plasmid carrying the tRNA and the Hsp82_D61C gene with an N-terminal GST- and C-terminal His-tag were co-transformed with the pBK plasmid carrying the respective PylRS^88^ into *E. coli* K12. Cells were grown in 2xYT (10 µg/mL tetracycline, 100 µg/mL ampicillin). At an OD_600_ of 0.6, 1 mM of the met-ncbK was added. At an OD_600_ of 0.8, protein expression was induced with 0.02% arabinose (4 h, 37 °C). Cells were harvested and resuspended in Ni-NTA buffer A (50 mM NaH_2_PO_4_, 500 mM NaCl, 10 mM imidazole, 1 mM DTT, pH 7.8) supplemented with EDTA-free protease inhibitor (SERVA) and DNase1. Cells were lysed using a cell disruption system (Constant Systems) at 1.8 kbar. After lysate clarification, the supernatant was loaded on a 5 mL HisTrap HP column (GE Healthcare), washed with 10 CV Ni-NTA buffer A and 10 CV 5% Ni-NTA buffer B (50 mM NaH_2_PO_4_, 500 mM NaCl, 300 mM imidazole, 1 mM DTT, pH 7.8). The bound proteins were eluted with 100% Ni-NTA buffer B. Fractions were diluted 1:10 with PBS (16 mM Na_2_HPO_4_, 4 mM KH_2_PO_4_ 115 mM NaCl, 2.7 mM KCL, pH 7.4) and loaded on a 5 mL GSTrap HP column (GE Healthcare), washed with 10 CV PBS, and eluted with GST buffer B (50 mM Tris, 20 mM reduced glutathione, pH 8.0). Fractions were incubated with TEV-protease ON at 4 °C. The solution was loaded on a Superdex 16/60 200 pg SEC column (GE Healthcare) and eluted with SEC buffer (40 mM HEPES, 150 mM KCl, 5 mM MgCl_2_).

To identify the incorporation efficiency of metK into Hsp82, Asp-N protease from *Pseudomonas fragi* (Promega) was used for digestion, prior to MALDI-MS analysis. The protein was digested according to the manufacturer’s protocol and MS analysis was conducted using a Biflex-II mass spectrometer (Bruker Daltonik, Bremen, Germany). Data were analysed using Mascot (Matrix Science, London, UK).

### Protein secondary structure

To determine the secondary structure of the purified mutants, Far-UV CD-spectra were measured. Proteins were dialyzed in 5 mM NaPO_4_ (pH 7.5) and a concentration of 0.2 mg/mL was used. CD-spectra were collected between 190 nm and 260 nm in a J-710 spectropolarimeter at 20 °C.

### ATPase assays

ATPase assays were performed by a spectrophotometric assay using an enzymatic ATP regenerating system^89^. 10 μM of human Hsp90 and 3 μM of yeast Hsp90 were used in 40 mM Hepes (pH 7.5), 150 mM KCl, 5 mM MgCl_2_. Measurements were performed at 30 °C for the yeast proteins and at 37 °C for the human homologues. To substract the background activity, 100 μM radicicol (Sigma, Germany) were added at the end of the measurement. The data were analyzed by linear regression using Origin 8.0 and for ATP-binding, data were fitted according to the Michaelis–Menten equation. For the activation by Aha1, a low salt assay buffer (40 mM Hepes (pH 7.5), 20 mM KCl, 5 mM MgCl_2_) was used, as the affinity between Hsp90 and Aha1 is salt sensitive^57^.

### Analytical ultracentrifugation (aUC)

To analyze the ability to form complexes with co-chaperones and clients and to determine structural changes, the Hsp90 mutants were subjected to aUC (Beckman, Krefeld, Germany). 0.5 μM of labelled co-chaperones (Aha1-FAM and Sba1-Atto488) were incubated in the absence (for Aha1 interaction) or presence of 2 mM AMP-PNP (for Sba1 interaction) with 3 μM of the respective Hsp90 mutants. Buffers were 40 mM Hepes (pH 7.5), 20 mM KCl, 5 mM MgCl_2_ for the Aha1 interaction and 40 mM Hepes (pH 7.5), 150 mM KCl, 5 mM MgCl_2_ for the Sba1 interaction. The buffer for human Hsp90 additionally contained 2 mM DTT. To analyze the interaction with the GR, 0.5 μM of Atto488-labelled GR-LBDm were used in 20 mM Hepes (pH 7.5), 20 mM KCl, 5 mM MgCl_2,_ 5 mM DTT and 2 mM ATP. For the experiments with yeast Hsp90 alone, 2 µM of Atto488-labelled Hsp82 in 40 mM Hepes (pH 7.5), 150 mM KCl, 5 mM MgCl_2_, incubated in the absence or presence of 5 mM AMP-PNP or 5 mM ATPγS were used. Runs were performed at 42,000 rpm, 20 °C and complex formation was monitored using an Avis Fluorescence detection system (Lakewood, USA). The raw data were converted into a dc/dt plot using SEDVIEW and curves were fitted by Gaussian equations.

### Yeast methods and FOA shuffling

The yeast strain ECU82α^62^, whose only copy of Hsp90 is on an URA3-containg pKAT6 plasmid, was used throughout this study and standard methods for growth and transformations were employed. Cells were cultured on selective minimal medium (0.67% yeast nitrogen base, 2% glucose and amino acids depending on auxotrophy) at 30 °C. To examine the ability of the human and yeast mutants to confer viability, constructs were subcloned into p423GPD vectors (ATCC^90^) and transformed into ECU82α. By a plasmid shuffling approach using 5-FOA, the pKAT6 plasmid was replaced with the yeast and human mutants, as the sole source of Hsp90 in the cell^91^.

### Yeast stress assays

To test the influence of the mutants, cells were challenged either by heat, UV irradiation, or the Hsp90 inhibitor radicicol. To investigate temperature effects, overnight cultures were adjusted to an OD_600_ of 0.5 and 1:5 dilutions were spotted on selective agar plates. Cells were incubated at the indicated temperatures for 48 h. To test the influence of Hsp90 in nucleotide excision repair, cells were spotted as described and plates were exposed to UV light (40 J/m^2^, 80 J/m^2^, or 120 J/m^2^) at room temperature and afterwards incubated for 48 h at 30 °C^28,67^. For the radicicol assay, overnight cultures were adjusted to an OD_600_ of 0.1 and the indicated amount of radicicol was added. Cells were grown overnight in the presence of the inhibitor at 30 °C and then spotted onto selective agar plates in 1:5 dilutions.

### GR activation assay in vivo

A sensitive readout for Hsp90 function in vivo is the activity of the mammalian GR receptor^62^. Yeast cells were transformed with a plasmid constitutively expressing GR. Positive clones were cultivated overnight in selective medium and diluted into media containing 10 μM desoxycorticosterone (Sigma, Germany) for ~12 h. As activated GR binds to a response element coupled to the β-galactosidase gene, the expression of β-galactosidase can be used as an indirect readout for GR activity. To monitor ß-galactosidase activity, cells were lysed and ONPG solution was added. The formation of the chromogenic substrate ortho-nitrophenol was monitored at 405 nm in a plate reader.

### v-src kinase assay

For the viral tyrosine kinase v-src, the assay was performed as previously described^62^. In short, a plasmid carrying v-src kinase under the inducible Gal promoter was transformed into the yeast cells. After the selection of positive transformants, cells were grown for 48 h in raffinose-containing medium before they were adjusted to the same OD and spotted in 1:5 dilutions onto glucose- and galactose-containing plates. The growth of the yeast was analyzed after 48 h at 30 °C.

### Molecular dynamics simulations

Atomistic molecular dynamics (MD) simulations were employed to study the WT and methylated Lys-594 models of Hsp90, as well as the in silico mutated variants K594R/E/I/A. The models were constructed based on the full-length yeast Hsp90 dimer in the closed state with the ATP-Mg^2+^ complex is obtained from the Protein Data Bank (PDB ID: 2CG9)^15^, and with missing loops modelled using MODELLER^92^. Each protein model was solvated in a water box with 100 mM NaCl concentration. The molecular systems comprised ~300,000 atoms and were simulated in an *NPT* ensemble at *T* = 310 K and *p* = 101.3 kPa for 200 ns using NAMD^93^, with a 2 fs integration timestep, and using the CHARMM27 force field^94^. Long-range electrostatics was treated using the particle mesh Ewald approach. RMSD and root-mean-square fluctuations of the protein backbone atoms were computed to probe simulation convergence and global Hsp90 dynamics. Visual molecular dynamics^95^ was used for analysis of the MD trajectories.

### Fluorescence resonance energy transfer (FRET)

FRET experiments were carried out, as described previously^16^. Hsp90 was labelled at position C61 with donor and acceptor dyes, respectively (ATTO 488 and ATTO-550, ATTO-TEC GmbH). Measurements were conducted at 30 °C in a Fluoromax 4 spectrometer. Two hundred nanomolars of labelled Hsp90 was used in 40 mM Hepes (pH 7.5), 150 mM KCl, 5 mM MgCl_2_, 2 mM DTT in the absence or presence of 2 mM AMP-PNP, 2 mM ATPγS or 2 mM ATP. The chase experiments were performed with a tenfold excess (4 µM) of unlabelled protein. Curves were fitted by a mono-exponential decay equation. For the K594metK variant, a bi-exponential fit was used taking into account the relative amounts of the methylated species. t1 was set fixed with the value of the wt protein.

1 $$y = 0.49 \cdot A1 \cdot {\mathrm{e}}^{\left( {\frac{{ - x}}{{t1}}} \right)} + 0.51 \cdot A1 \cdot {\mathrm{e}}^{\left( {\frac{{ - x}}{{t2}}} \right)} + y0.$$

### Hsp90 dimerization assay

Measurements were conducted at 25 °C in a Shimadzu HPLC using a Superdex 200 increase 10/300 GL in 40 mM Hepes (pH 7.5), 150 mM KCl, 5 mM MgCl_2_, and 2 mM DTT. Concentration of human Hsp90β varied from 2 nM to 4 µM. Concentration of yeast Hsp90 varied from 16 nM to 2 µM. Dimerization assays were performed similar to Richter et al.^43^. In short, a dilution series of each protein variant was applied, using a volume of 200 µL. Fluorescence was detected via a RF-10Axl detector (Shimadzu) with excitation at 280 nm and emission at 320 nm wavelength. The gain of the photomultiplier was adopted for the different concentrations over the time course of the measurement. Data were fitted, using Origin 8.0 via the following equation:

2 $${\mathrm{ET}} = {\mathrm{ET}}_{{\mathrm{mono}}} - ({\mathrm{ET}}_{{\mathrm{mono}}} - {\mathrm{ET}}_{{\mathrm{dimer}}}) \cdot \frac{{[{\mathrm{protein}}]}}{{\left[ {{\mathrm{protein}}} \right] + K_D(app)}}.$$

ET represents the elution time, ET_mono_ the elution time of the Hsp90 monomeric species, and ET_dimer_ the elution time of the Hsp90 dimer. For the determination of the K_D_, the K_D_ (app) was divided by the dilution factor, which is the quotient of the theoretical width of the peak at the point of injection (0.2 mL/0.5 mL/min = 0.4 min) and the observed peak width and was about 4 for both Hsp82 and human Hsp90.

### Reporting summary

Further information on research design is available in the Nature Research Reporting Summary linked to this article.

## Supplementary information

Supplementary Information

Reporting Summary

## Data Availability

The source data underlying Figs. 1c, 3a–c, 4a–c, and 6c are provided as a Source Data file. Other data are available from the corresponding author upon reasonable request.

## Source Data

Source Data
