# Author Correction: A methylated lysine is a switch point for conformational communication in the chaperone Hsp90

**DOI:** 10.1038/s41467-020-17621-7

**Published:** 2020-07-21

**Authors:** Alexandra Rehn, Jannis Lawatscheck, Marie-Lena Jokisch, Sophie L. Mader, Qi Luo, Franziska Tippel, Birgit Blank, Klaus Richter, Kathrin Lang, Ville R. I. Kaila, Johannes Buchner

**Affiliations:** 1 Department of Chemistry, Technische Universität München, Lichtenbergstr. 4, 85747 Garching, Germany; 2 Institute for Advanced Study, Lichtenbergstraße 2a, 85748 Garching, Germany; 3 Soft Matter Research Center and Department of Chemistry, Zhejiang University, Zhejiang, 310027 PR China; 4 Department of Biochemistry and Biophysics, Stockholm University, SE-106 91 Stockholm, Sweden; 5 Present Address: Institut für Mikrobiologie der Bundeswehr, Neuherbergstr.11, 80937 München, Germany; 6 Present Address: NanoTemper Technologies GmbH, Flößergasse 4, 81369 München, Germany; 7 Present Address: Max Planck Institut für Biochemie, Department Molekulare Medizin, Am Klopferspitz 18, 82152 Martinsried, Germany

**Keywords:** Chaperones, Chaperones

Correction to: *Nature Communications* 10.1038/s41467-020-15048-8, published online 5 March 2020.

In the original version of the manuscript’s Supplementary Information, Supplementary Figure 2 was inadvertently duplicated and reproduced as Supplementary Figure 3. The error has now been fixed and the corrected Supplementary Information PDF is available to download.

