# Supplementary material for: A methylated lysine is a switch point for conformational communication in the chaperone Hsp90
Source: Nat Commun. 2020 Mar 5;11:1219. doi: 10.1038/s41467-020-15048-8 (PMC7057950; doi:10.1038/s41467-020-15048-8)
Supplement: Supplementary file 1 — Supplementary Information [file 41467_2020_15048_MOESM1_ESM.pdf]

Supplementary Information

A methylated lysine is a switch point for conformational communication in the chaperone Hsp90

Alexandra Rehn, Jannis Lawatscheck et al.

Supplementary Figure 1

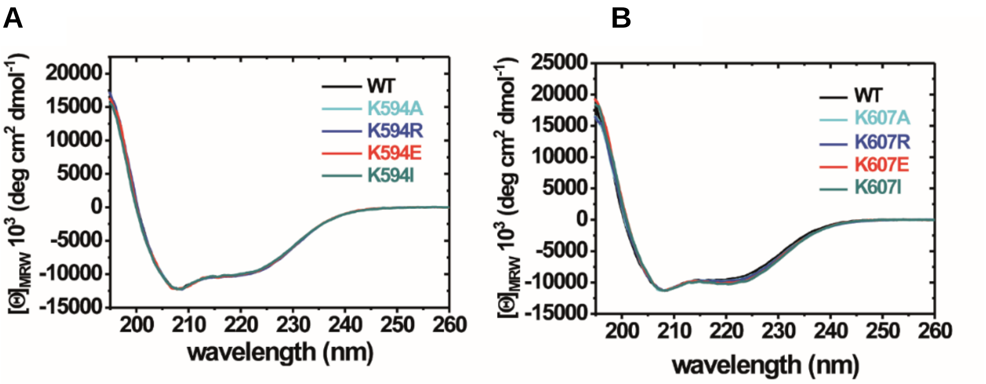

Supplementary Figure 1: Secondary structure of the different Hsp90 mutants.

Secondary structure of the different yeast (A) and human (B) mutants monitored via CD-spectroscopy.

Supplementary Figure 2

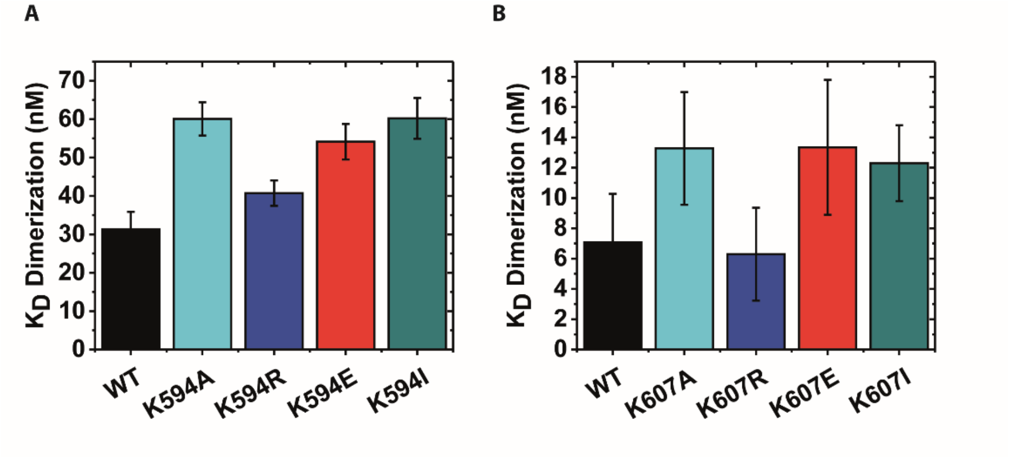

Supplementary Figure 2: Dissociation constants for the dimer formation of the different Hsp90 mutants.

Dissociation constants (K_D) for the dimer formation of the different yeast (A) and human (B) mutants. Error bars represent s.d. of the individual Data-points from the calculated fit for a single measurement (n = 1).

Supplementary Figure 3

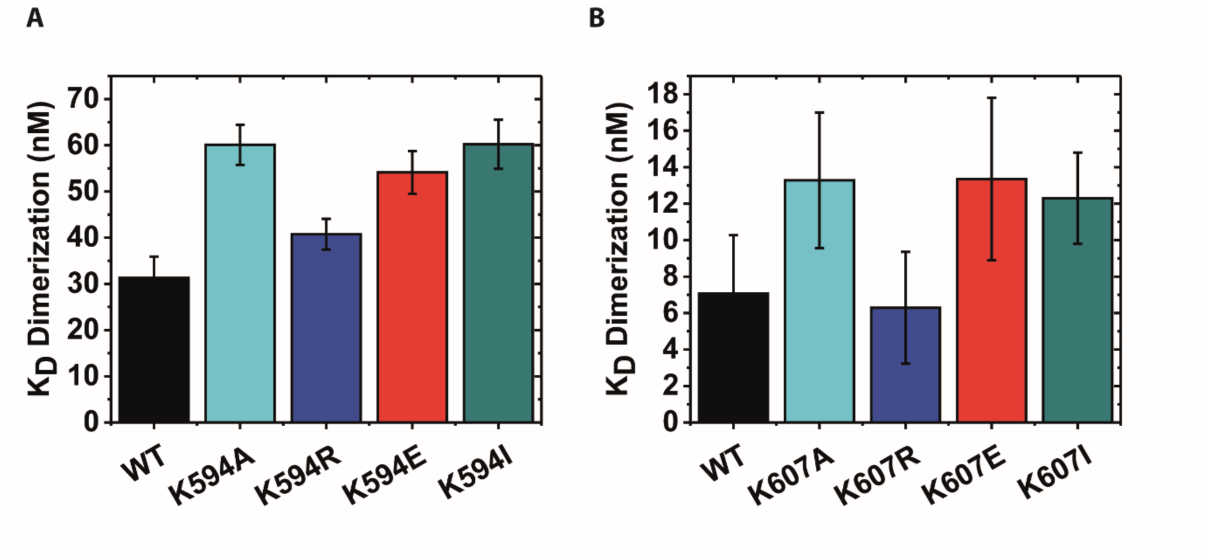

Supplementary Figure 3: ATP binding of the different Hsp90 mutants.

ATP binding was determined using ATPase activity assays. The ATP turnover was applied against the respective ATP concentrations (A). A Michaelis Menten fit was applied and K_M-values were compared (B). Error bars represent s.d. of the individual Data-points from the calculated fit for a single measurement (n = 1).

Supplementary Figure 4

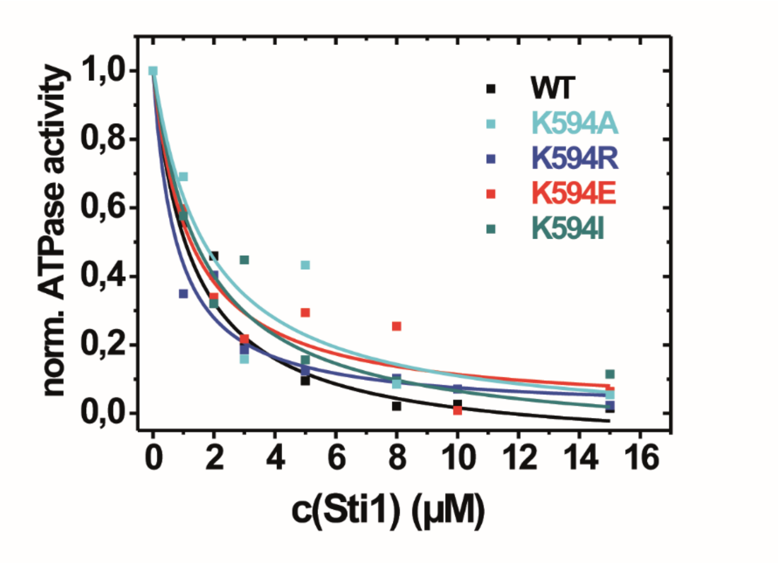

Supplementary Figure 4: Effect of Sti1 on the ATPase activity of the different Hsp90 mutants.

Normalized ATPase activity of the Hsp90 mutants in the presence of the inhibitory protein Sti1.

Supplementary Figure 5

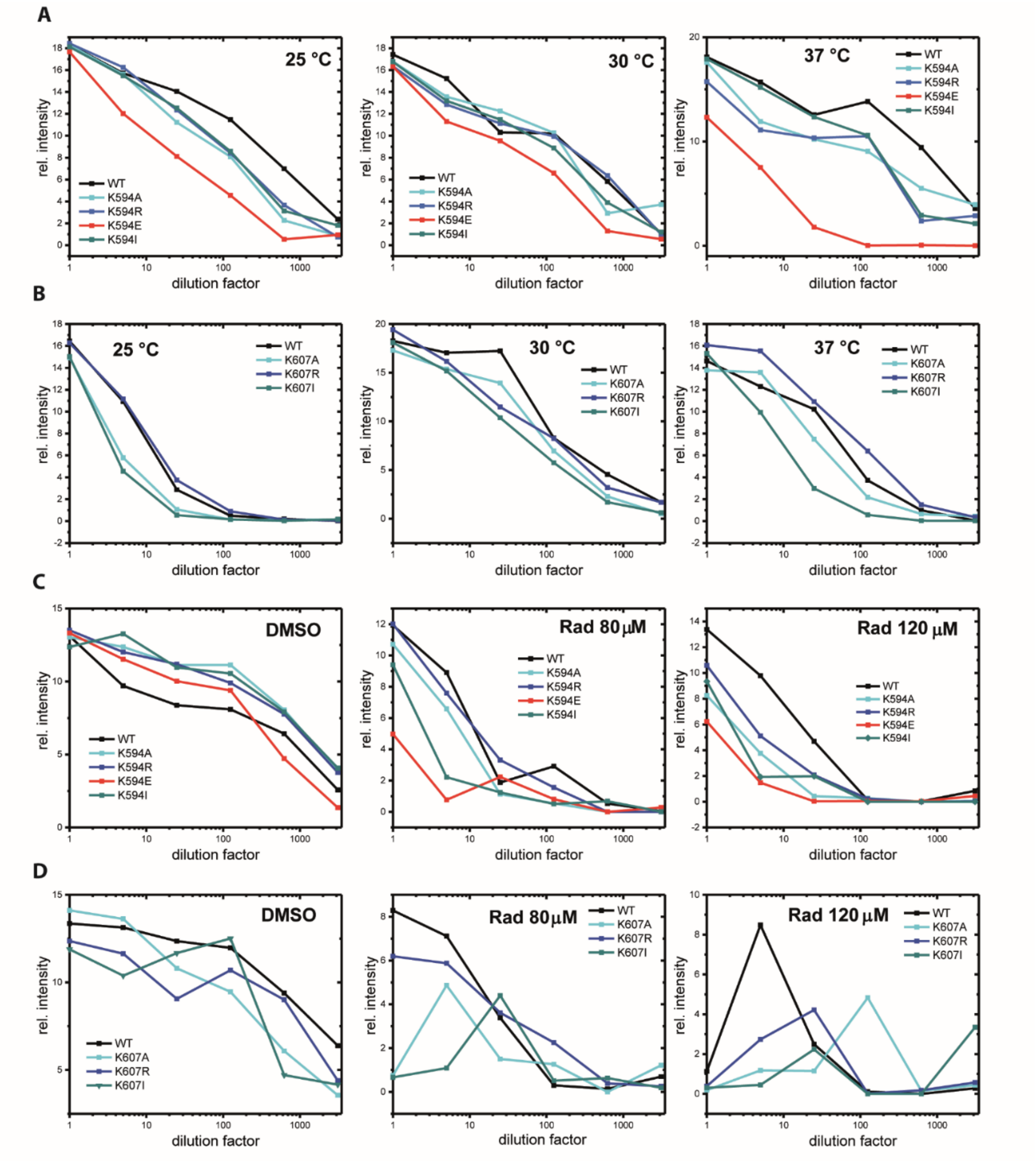

Supplementary Figure 5: Quantification of drop assays (Figure 3B,C)

Quantification of drop assays to observe growth differences between the different strains containing the yeast Hsp90 (A) and human Hsp90 (B) variants. All shuffled yeast strains grew similarly at 30°C but started showing growth deficiencies at lower and higher temperatures. Yeast Hsp90 (C) and human Hsp90 (D) mutants were tested towards their sensitivity against various concentrations of the inhibitor radicicol.

Supplementary Figure 6

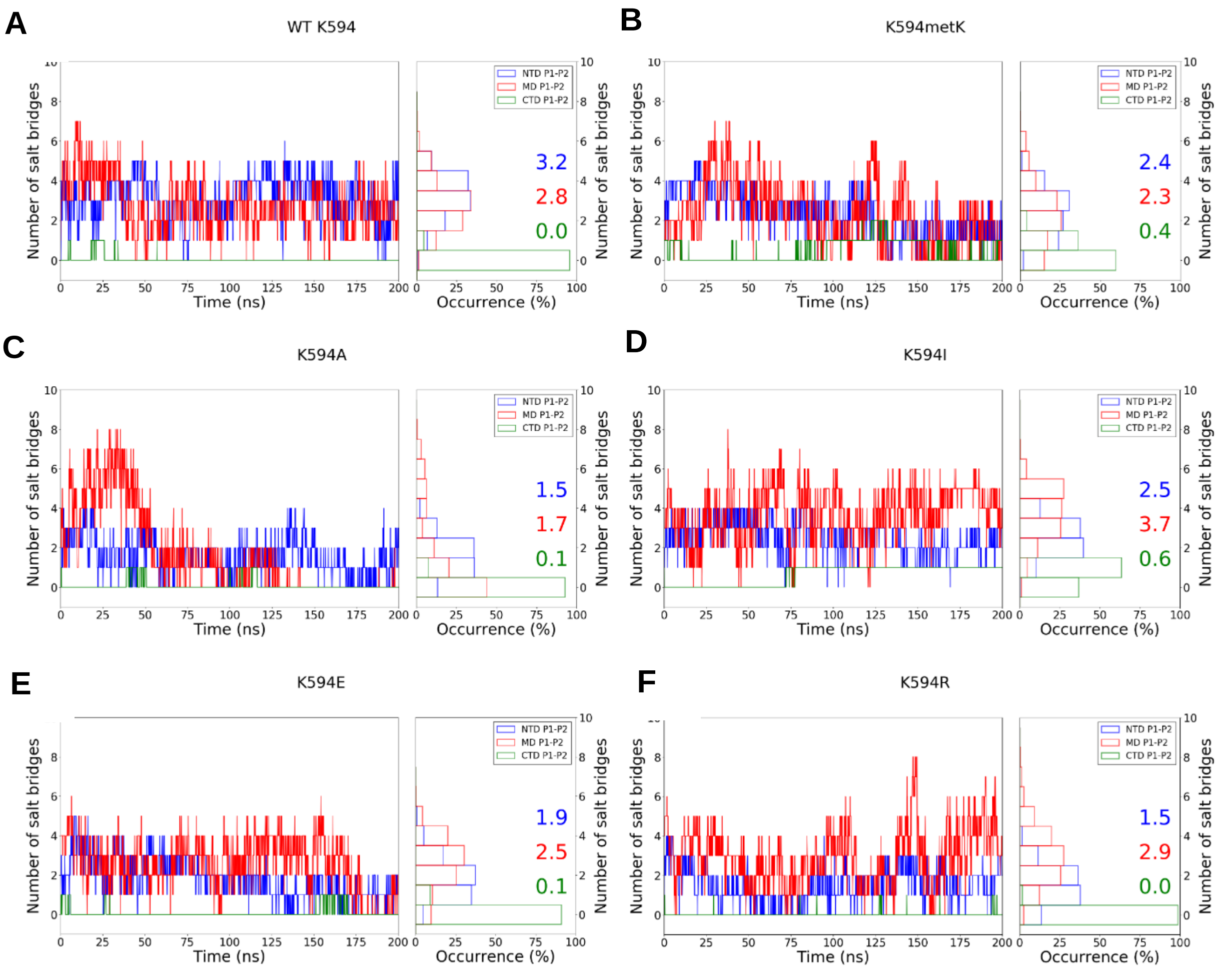

Supplementary Figure 6: Modulation of the ion-pair dynamics between different domains of Hsp90 by Lys-594.

(A – F) Average number and distributions of salt bridges between the CTD (green), middle domain (red), and NTD (blue) between the Hsp90 protomers (here P1 and P2) in the WT, mono-methylated Lys-594, and the different mutants. The mean distributions of the ion pair in the CTD/MD/NTD are given in green/red/blue, respectively.

Supplementary Figure 7

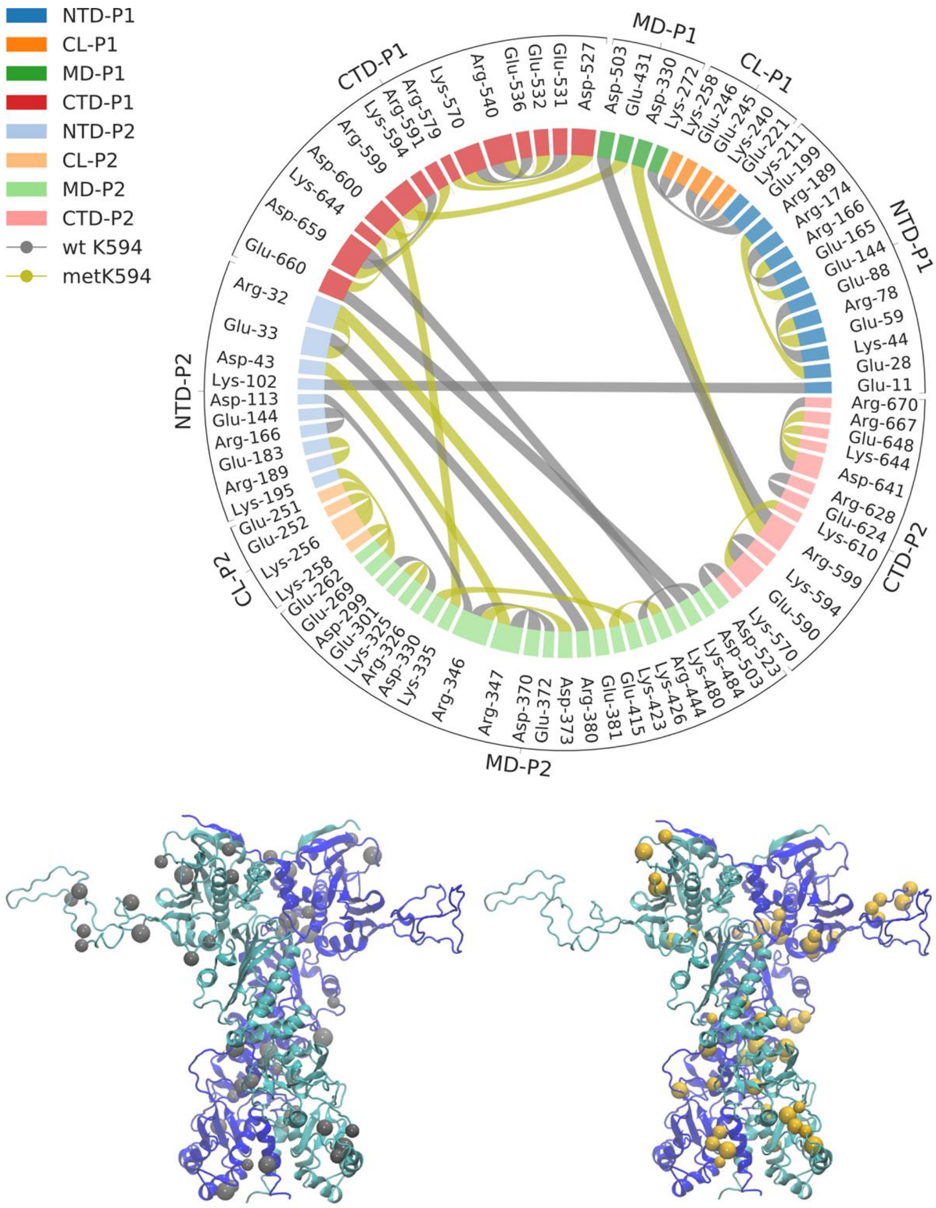

Supplementary Figure 7: Chord plot of wild type yeast Hsp90 and the metK594 variant.

Salt bridges in wild type Hsp90 (in gray) and the metK594 variant (in yellow) during 200 ns atomistic MD simulations. To focus on the differences between the two forms, only contacts that are present in >67% higher interaction probability in one form relative to the other one are shown. The two protomers of the full-length dimer are divided into N-terminal domain (NTD, blue), the charged linker region (CL, orange), the middle domain (MD, green), and the C-terminal domain (CTD, red). Amino acids involved in interactions in wild type Hsp90 (left) and the metK594 variant (right) are shown in the bottom part of the figure.

Supplementary Figure 8

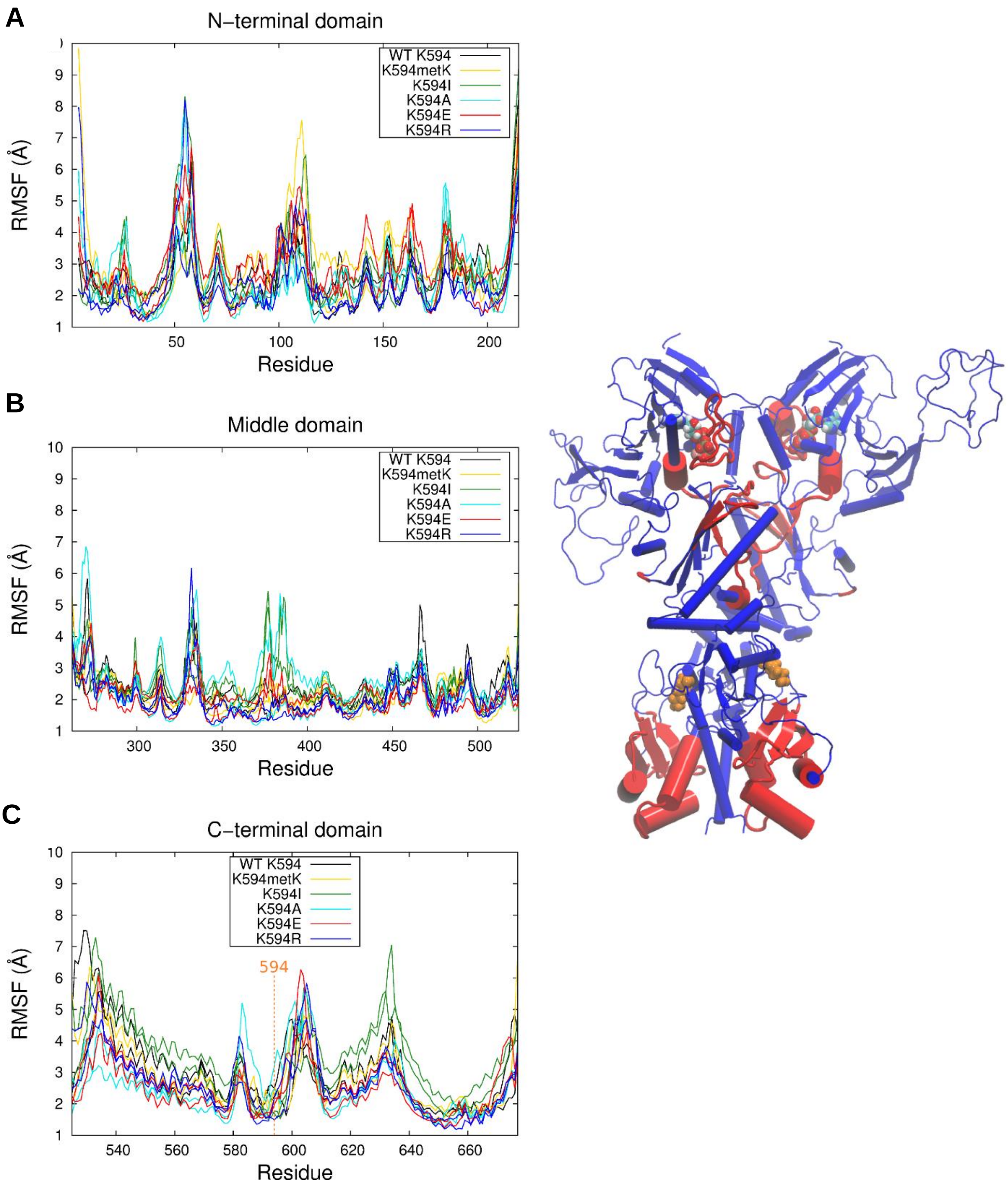

Supplementary Figure 8: Root-mean-square-fluctuations (RMSF) of backbone atoms in Hsp90 during MD simulations.

(A – C) RMSF profiles for the NTD (A), middle domain (B), CTD (C) for WT Hsp90, mono-methylated Lys-594 and the different mutants. The RMSFs are plotted for each protomer to reflect the statistical uncertainty of the data. *Inset*: The areas marked in red on the WT Hsp90 structure (D) [1] have an increased backbone RMSF in the K594I mutant as compared to the WT Hsp90. Lys-594 is shown in an orange vdW representation.

Supplementary Figure 9

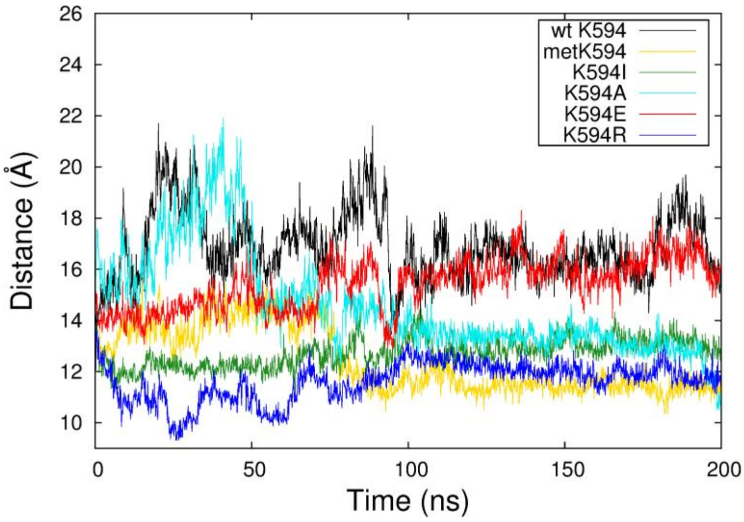

Supplementary Figure 9: Distance between helix 1 at the dimerization interface of the CTD in WT Hsp90 and upon K594 modification.

The K594I and K594R substitutions resemble the methylated K594 (metK594), with closer distances between the CTDs (*cf*. also Figure 5).

Supplementary Figure 10

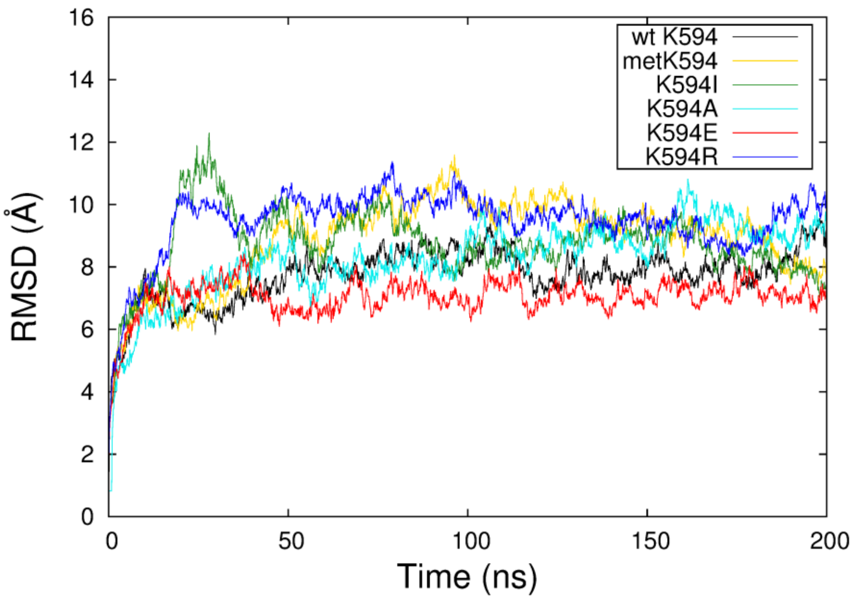

Supplementary Figure 10: Root-mean-square-deviation (RMSD) of the different Hsp90 mutants during MD simulations.

The RMSD was computed over all backbone atoms relative to the X-ray structure of the yeast Hsp90 [1].

Supplementary Figure 11

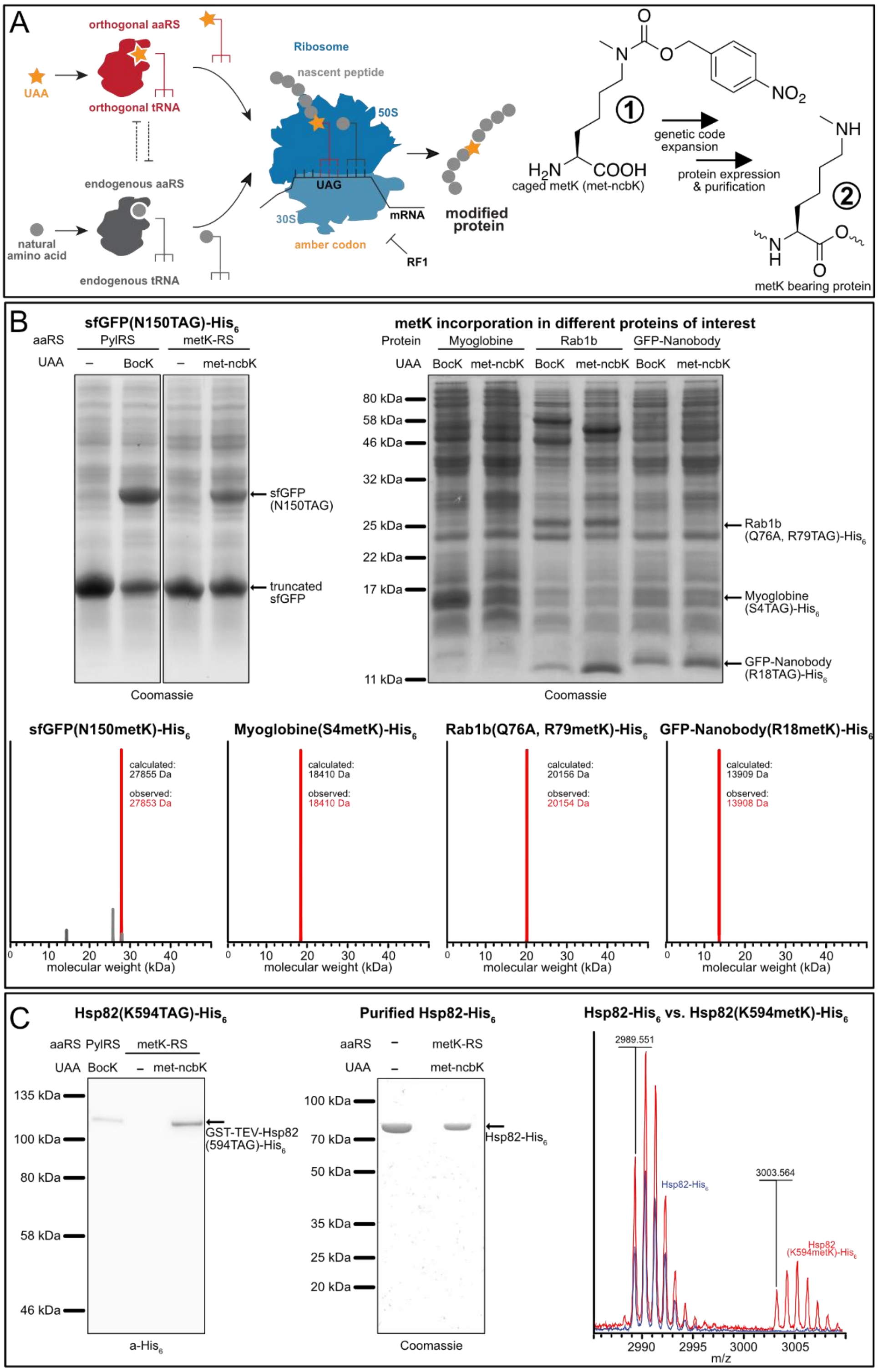

Supplementary Figure 11: Site-specific incorporation of N^6-monomethyl-L-lysine (metK) into proteins via genetic code expansion

(A) Genetic code expansion: To site-specifically incorporate an unnatural amino acid (UAA) (yellow star) into a protein of interest, an orthogonal aminoacyl-tRNA synthetase (aaRS)/tRNA pair (red) is needed, which recognizes the UAA and charges is onto an orthogonal tRNA (red) that directs the incorporation of the UAA according to an amber codon in the mRNA. Since monomethylation at the ε-amino group of lysine is a very small post-translational modification (PTM), metK is recognized by the lysine-aaRS and cannot specifically be introduced into proteins[2-6]. To circumvent this, we used N^6-methyl-N^6-(((4-nitrobenzyl)oxy)carbonyl)-L-lysine (met-ncbK), which gets decaged to metK during protein purification due to reduction of the aromatic nitro group, followed by fragmentation [7]. (B) Site-specific incorporation of metK into proteins**:** The SDS-PAGE on the left shows amber suppression with and without Me-nocK in comparison to Boc-lysine (BocK). The right gel shows site-specific incorporation of met-ncbK in comparison to BocK into Myoglobine (Myoglobine(S4TAG)-His$_6$), Rab1b (Rab1b(Q76A, R79TAG)-His$_6$) and GFP-nanobody (GFP-nanobody(R18TAG)-His$_6$), bearing site-specific amber codons, respectively. Full-length ESI-LC MS of all four proteins confirms site-specific incorporation of met-ncbK and quantitative decaging to metK. (C) Site-specific incorporation of metK into Hsp82**:** The western blot on the left shows the expression level after amber suppression of GST-TEV-Hsp82(K594TAG)-His$_6$ incorporating BocK or met-ncbK, respectively. The SDS-PAGE (middle) shows the purified WT protein (Hsp82-His$_6$) in comparison to the mono-metylated protein (Hsp82(K594metK)-His$_6$). On the right, MALDI-TOF analysis of the Asp-N digested Hsp82-His$_6$ (blue) and the Hsp82(K594metK)-His$_6$ (red). The respective peptide, containing K594 (DAPAAIRTGQFGWSANMERIM**K**AQALR MW: 2989.5247 Da) was found. In the case of Hsp82(K594metK)-His$_6$, a shift of 14 Da was detected, showing an about 30% incorporation of metK.

Supplementary Table 1

S-Values for WT yeast Hsp90, the K594I and K594E mutants in the presence of different nucleotides.

	WT	K594I	K594E
**w/o**	5.2	5.3	5.2
**ATPγS**	5.7	5.6	5.3
**AMP-PNP**	5.9	5.4	5.3

Supplementary Table 2

Primer Sequences used in this study.

	**Fwd**	**Rev**
**K594A**	5'-AAGAATCATGgcgGCTCAAGCCTTG-3'	5'-TCCATGTTAGCAGACCAAC-3'
**K594R**	5'-AAGAATCATGcgtGCTCAAGCCTTGAG-3'	5'-TCCATGTTAGCAGACCAAC-3'
**K594E**	5'-AAGAATCATGgaaGCTCAAGCCTTG-3'	5'-TCCATGTTAGCAGACCAAC-3'
**K594I**	5'-AAGAATCATGattGCTCAAGCCTTGAG-3'	5'-TCCATGTTAGCAGACCAAC-3'
**K594Amber**	5'-AAGAATCATGtagGCTCAAGCCTTG-3'	5'-TCCATGTTAGCAGACCAAC-3'
**K607A**	5'-GCGGATCATGgcgGCCCAGGCAC-3'	5'-TCCATATTGGCTGTCCAG-3'
**K607R**	5'-GCGGATCATGcgtGCCCAGGCAC-3'	5'-TCCATATTGGCTGTCCAG-3'
**K607E**	5'-GCGGATCATGgaaGCCCAGGCAC-3'	5'-TCCATATTGGCTGTCCAGCC-3'
**K607I**	5'-GCGGATCATGattGCCCAGGCAC-3'	5'-TCCATATTGGCTGTCCAG-3'

Supplementary References

1 Ali, M. M. *et al.* Crystal structure of an Hsp90-nucleotide-p23/Sba1 closed chaperone complex. *Nature* **440**, 1013-1017, doi:10.1038/nature04716 (2006).

2 Ai, H. W., Lee, J. W. & Schultz, P. G. A method to site-specifically introduce methyllysine into proteins in E. coli. *Chem Commun (Camb)* **46**, 5506-5508, doi:10.1039/c0cc00108b (2010).

3 Groff, D., Chen, P. R., Peters, F. B. & Schultz, P. G. A genetically encoded epsilon-N-methyl lysine in mammalian cells. *Chembiochem* **11**, 1066-1068, doi:10.1002/cbic.200900690 (2010).

4 Nguyen, D. P., Garcia Alai, M. M., Kapadnis, P. B., Neumann, H. & Chin, J. W. Genetically encoding N(epsilon)-methyl-L-lysine in recombinant histones. *J Am Chem Soc* **131**, 14194-14195, doi:10.1021/ja906603s (2009).

5 Wang, Y. S. *et al.* A genetically encoded photocaged Nepsilon-methyl-L-lysine. *Mol Biosyst* **6**, 1557-1560, doi:10.1039/c002155e (2010).

6 Yanagisawa, T. *et al.* Multiple site-specific installations of Nepsilon-monomethyl-L-lysine into histone proteins by cell-based and cell-free protein synthesis. *Chembiochem* **15**, 1830-1838, doi:10.1002/cbic.201402291 (2014).

7 Virdee, S. *et al.* Traceless and site-specific ubiquitination of recombinant proteins. *J Am Chem Soc* **133**, 10708-10711, doi:10.1021/ja202799r (2011).
